# Ginsenoside Rb1 protects human vascular smooth muscle cells against resistin-induced oxidative stress and dysfunction

**DOI:** 10.3389/fcvm.2023.1164547

**Published:** 2023-05-25

**Authors:** Weifeng Lu, Yue Lin, Nezam Haider, Pricila Moly, Lixin Wang, Wei Zhou

**Affiliations:** ^1^ Department of Vascular Surgery, Zhongshan Hospital (Xiamen), Fudan University, Xiamen, China; ^2^ Division of Vascular Surgery, Department of Surgery, University of Arizona, Tucson, AZ, United States; ^3^ Department of Vascular Surgery, Zhongshan Hospital, Fudan University, Shanghai, China

**Keywords:** ginsenoside Rb1, resistin, vascular smooth muscle cells (VMSCs), reactive oxygen species(ROS), superoxide dismutase(SOD)

## Abstract

Resistin has been shown to play a key role in inducing vascular smooth muscle cells (VSMCs) malfunction in the atherosclerosis progression. Ginsenoside Rb1 is the main component of ginseng, which has been used for thousands of years and has been reported to have a powerful vascular protective effect. The aim of this study was to explore the protective effect of Rb1 on VSMCs dysfunction induced by resistin. In the presence or absence of Rb1, human coronary artery smooth muscle cells (HCASMC) were treated at different time points with or without 40 ng/ml resistin and acetylated low-density lipoprotein (acetylated LDL). Cell migration and proliferation were analyzed using wound healing test and CellTiter Aqueous Cell Proliferation Assay (MTS) test, respectively. Intracellular reactive oxygen species (ROS) (H2DCFDA as a dye probe) and superoxide dismutase (SOD) activities were measured by a microplate reader and the differences between groups were compared. Rb1 significantly reduced resistin-induced HCASMC proliferation. Resistin increased HCASMC migration time-dependently. At 20 µM, Rb1 could significantly reduce HCASMC migration. Resistin and Act-LDL increased ROS production to a similar level in HCASMCs, while Rb1 pretreated group reversed the effects of resistin and acetyl-LDL. Besides, the mitochondrial SOD activity was significantly reduced by resistin but was restored when pretreated with Rb1. We confirmed the protection of Rb1 on HCASMC and suggested that the mechanisms involved might be related to the reduction of ROS generation and increased activity of SOD. Our study clarified the potential clinical applications of Rb1 in the control of resistin-related vascular injury and in the treatment of cardiovascular disease.

## Introduction

Ginseng is a traditional herbal adaptation, which has a history of more than 5,000 years. It is widely used in Asia and also in western medicine. At present, it is simply made into a drink to improve physical performance and treat many chronic diseases, such as cancer and cardiovascular, inflammatory, neuronal, and metabolic diseases (1). In the United States of America, ginseng ranks second and fifth in use among adult best-selling natural products in 2002 and 2007, respectively (2). Ginsenoside, a sterol glycoside, is found only in ginseng, which are divided into two groups: Rb1 group and Rg1 group. Ginsenoside Rb1 (Rb1) is the main active components from ginseng, the pharmacological effects of which attract much attention in the endocrine, cardiovascular, and immune systems in recent years (3–9).

Cardiovascular diseases (CVD) are now one of the leading causes of disability and death in the world, especially in the elderly population. Recently, accumulating evidence has proven that Rb1 demonstrated its cardioprotective effect both *in vitro* and *in vivo*. We first reported that Rb1 could block homocysteine-induced endothelial dysfunction and superoxide anion production effectively in porcine coronary arteries by *in vitro* studies (5). It was reported that Rb1 could also protect human endothelial cells from oxidized low-density lipoprotein (oxLDL) (6). In vivo studies showed that pretreatment with Rb1 reduced cardiomyocyte apoptosis induced by myocardial ischemia/reperfusion injury in diabetic rats and alleviated cardiac dysfunction (7). In a rat model, Rb1 was proved to inhibit the carotid intima hyperplasia induced by balloon dilation (8). Rb1 administration promoted atherosclerotic plaque stability along with increased macrophage autophagy and M2 phenotype in the atherosclerosis model in ApoE^−/−^ mice (4, 9). However, few studies examined the protective effect of Rb1 on vascular smooth muscle cells (VSMCs), especially on human VSMCs.

The key feature of vascular proliferation diseases is the abnormal proliferation and migration of VSMC, including atherosclerosis, restenosis after vascular injury and vascular wall hypertrophy caused by hypertension.(10–12) Resistin is an adipokine mainly expressed in human monocyte/macrophage lineage cells, which is closely related to cardiovascular diseases and adverse clinical outcomes (13). Resistin has been proved to act in a variety of cells including VSMCs, indicating that resistin has a certain role in atherosclerosis (14). We and others demonstrated that resistin at a pathological concentration promotes vascular smooth muscle cell (VSMC) proliferation and migration, as well as reactive oxygen species (ROS) production (15–17). We also demonstrated that inflammation was induced in VSMCs and VSMCs dysfunction by resistin through protein kinase C epsilon (PKC*ε*)-mediated NADPH oxidase (Nox) activation (17). However, the role of Rb1 in VSMCS dysfunction and related mechanisms by resitin has not been investigated and needs further confirmation. Considering the protective role of Rb1 in cardiovascular disease, we hypothesized that Rb1 may protect the progression of atherosclerosis by reversing resistin induced VSMCs dysfunction and oxidative stress. To test the hypothesis, we tested the proliferation and migration of VSMCs induced by resistin with or without Rb1. We also validated the related mechanism was associated with enhancing antioxidation property of Rb1 by reducing ROS production and increasing superoxide dismutase (SOD) activity in VSMCs.

## Methods

### In vitro treatment and cell culture

Experiments were performed using human coronary artery smooth muscle cells (HCASMC) or VSMCs from Genlantis (18) at passage 5–7. Before each experiment, the medium was replaced to Dulbecco’s Modified Eagle Medium (DMEM) supplemented with 2% FBS for at least 8 h (hs) to starve the cells. In our study, we selected a pathological resistin level (40 ng/ml) which based on the published data of resistin in human subjects and our previous study, indicating that this concentration leads to VSMCs dysfunction and intimal hyperplasia (17, 19). Cells were managed with or without resistin at 40 ng/ml in the presence or absence of ginsenoside Rb1 (G0777-5MG, Sigma-Aldrich) for various time points at 20 µM according to previous research (*n* = 5) (4). Acetylated low-density lipoprotein (acetyl-LDL) was also used to treat cells in proliferation and ROS experiments because it is known to accumulate in macrophages to form foam cells and play a critical role in the mechanism of atherosclerosis by stimulating the proliferation of VSMCs (20).

### Cell proliferation assay

The cells treated with resistin (40 ng/ml) or acetyl-LDL (20 µg/ml) in the presence or absence of Rb1 (20 µM) for 1 h were seeded in a 96-well plate (5,000 cells/well) and incubated for an extra 24 h. Cell proliferation was evaluated by the usage of CellTiter 96® AQueous One Solution Cell Proliferation Assay (MTS assay, Promega). Briefly, 20 ml MTS was added to the cells in each well containing the samples in 100 µl of culture medium and cultured for 4 h at 37°C in a humidified 5% CO_2_ atmosphere. The cell plate covered with tinfoil was then subjected to a plate reader to fetch the absorbance at 490 nm. The formula of cell viability was calculated as follows: Cell Viability = (OD_sample_−OD_background_)/(OD_control_−OD_background_) × 100%.

### Cell migration assay

VSMCs migrations were evaluated by wound healing assay and transwell assay. VSMCs were grown and converged in a 6-well plate. A transverse scratch wound on each monolayer of VSMC was made by using a sterilized 200 µl-tip. The scratched VSMCs were then stimulated with or without resistin (40 ng/ml) in the presence or absence of Rb1 (20 µM) for an additional 6, 18, and 24 h, then the transverse scratch wounds were reexamined for cell migration. Pictures were captured with a phase-contrast microscope, and cell migration was quantified using ImageJ software, which was calculated as the percent of the wound closure area relative to that at the start point (*t* = 0).

Transwell assay was performed in Transwell chambers (HTS Transwell-96 Well Plate, 5 µm pore size; Corning Inc., USA). Briefly, 1 × 10^5^/ml cells were seeded on the Transwell Inserts in 75 µl of serum-free medium with or without Rb1 (20 µM). The bottom plate contained 235 µl of complete growth medium with resistin at 40 ng/ml. Cells migrated at 37°C for 24 h. After 24 h of culture, the non-migrated cells on the membrane top surface were removed with a cotton swab. After that, the migrated cells on the bottom side of the membrane were stained with 0.1% crystal violet and then destained with PBS.

### Intracellular ROS evaluation

Intracellular ROS were evaluated using the fluorescent probe 2′,7′-dichlorodihydrofluorescein diacetate (H_2_DCFDA) (ab113851, Abcam) according to the supplier's protocol. Briefly, cells were seeded in a 96-well plate (2.5 × 10^4^/well) overnight, then incubated with the probe (25 µM final concentration) for 45 min at 37°C in a humidified, 5% CO_2_ atmosphere. Then the cells were washed in PBS and treated with or without resistin or acetyl-LDL in supplemented buffer containing 10% fetal bovine serum (FBS) for 2 h. Rb1 was added at the same time (Co-treatment group) or 24 h before the addition of resistin or acetyl-LDL (Pre-treatment group). The pretreatment group was set because we found the exposure time of 2 h was not enough for Rb1 to take effect. The fluorescence of the cell homogenate was measured on a fluorescence plate reader at Ex/Em = 485/535 nm at the end point. The data are expressed as the ratio of fluorescence/fluorescence of the unstimulated control. Tert-Butyl Hydrogen Peroxide (TBHP) was used as a positive control.

### Measurement of antioxidant enzyme activity

In order to elucidate the effects of Rb1 after resistin exposure, SOD activities in the homogenate were measured by an enzymatic assay using a commercial kit (706,002, Cayman Chemical) according to the manufacturer's instructions. In brief, cells seeded in a 6-well plate were pretreated with Rb1 for 24 h and then exposed to resistin for 2 h. The cells in each group were harvested using a rubber policeman and sonicated in cold 20 mM Hepes (PH 7.2, containing 1 mM EGTA, 210 mM mannitol, and 70 mM sucrose). Then the cell lysates were used for assaying total SOD activity (cytosolic and mitochondrial). To separate these two enzymes, the cell lysates were centrifugated at 10,000×g for 15 min at 4°C. The resulting 10,000×g supernatant contained cytosolic SOD and the pellet contained mitochondrial SOD. For SOD assay, 200 µl of radical detector and 10 µl of SOD standard with different concentrations or 10 µl of sample were added to each designated well. The reactions were initiated by adding 20 µl of xanthine oxidase to all wells except the sample background. The 96-well plate was incubated on a shaker for 30 min at room temperature. The absorbance was read at 450 nm by a plate reader. The SOD activity of the samples was calculated from the linear regression of the standard curve. One unit of SOD is defined as the amount of enzyme needed to exhibit 50% dismutation of the superoxide radical.

### Statistical analysis

The numeric data are presented as mean ± SD. Differences between groups were analyzed by one-way ANOVA or an independent sample *t*-test. If the *p*-value is less than 0.05, the results are considered to be statistically different.

## Results

### Rb1 ameliorated resistin-stimulated HCASMC proliferation

**Figure 1:**
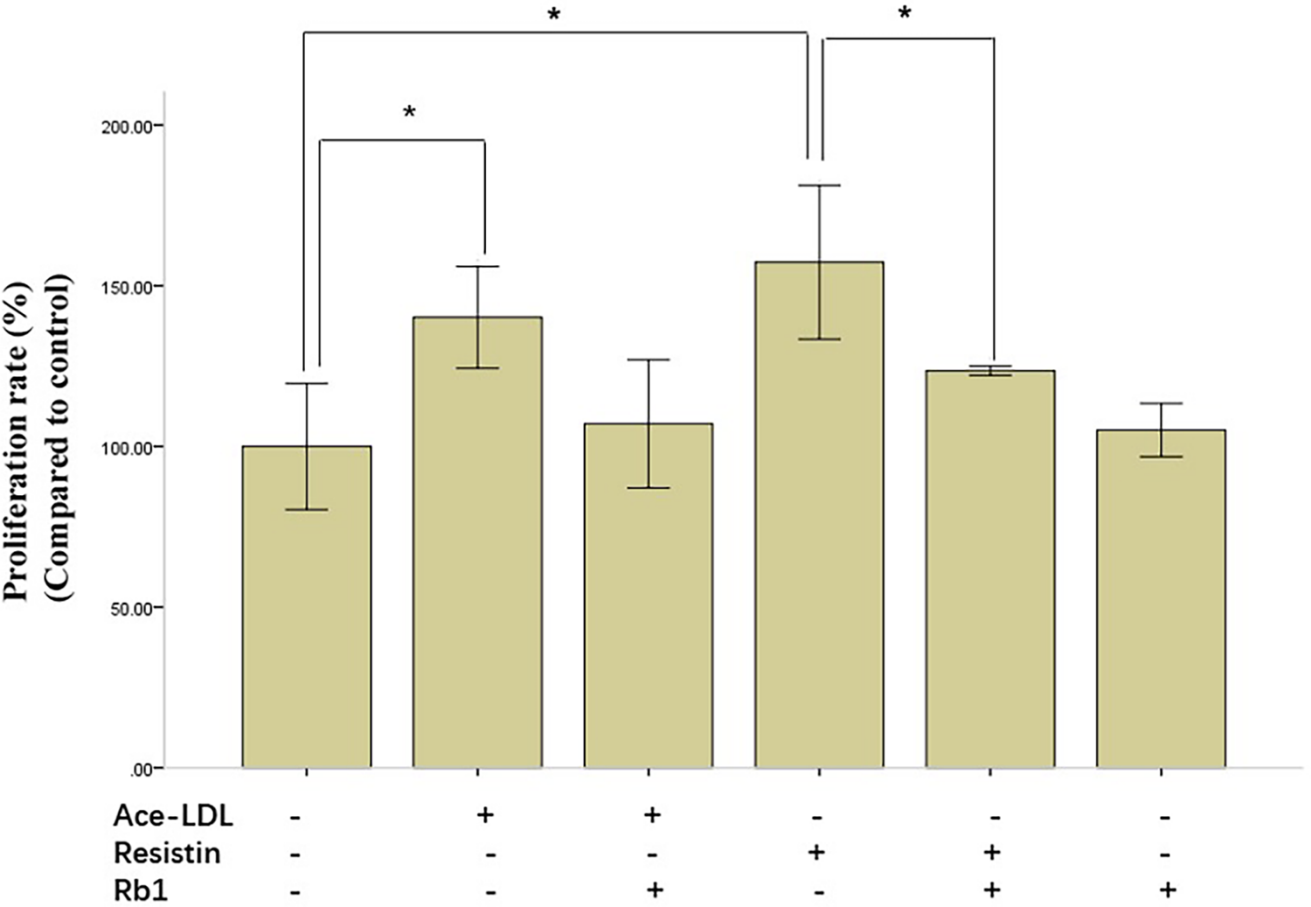
Resistin-induced human coronary artery smooth muscle cells (HCASMC) proliferation was ameliorated by Rb1. HCASMC proliferation, evaluated by MTS assay, was significantly increased by resistin and acetyl-LDL, which could be restored by Rb1. Note that Rb1 alone had no effect on proliferation of HCASMC. Cell Viability = (ODs−ODb)/(ODc−ODb) × 100%. OD_s_, the OD value of the samples; OD_b_, the OD value of the background; OD_c_, the OD value of the negative control. Values were means and SD (*n* = 5). *P < 0.05.

We first examined the effect of Rb1 on resistin-stimulated HCASMC proliferation by MTS assay. Data on the percentage of viable cells after treatment revealed that both resistin and acetyl-LDL stimulated HCASMC proliferation significantly. Rb1 alone had no effect on the proliferation of HCASMC, however, it significantly reduced resistin-induced HCASMC proliferation (Figure 1). Rb1 also ameliorated acetyl-LDL induced proliferation of HCASMC, although no statistical significance had been found in our data.

### Rb1 suppressed resistin-induced HCASMC migration

**Figure 2:**
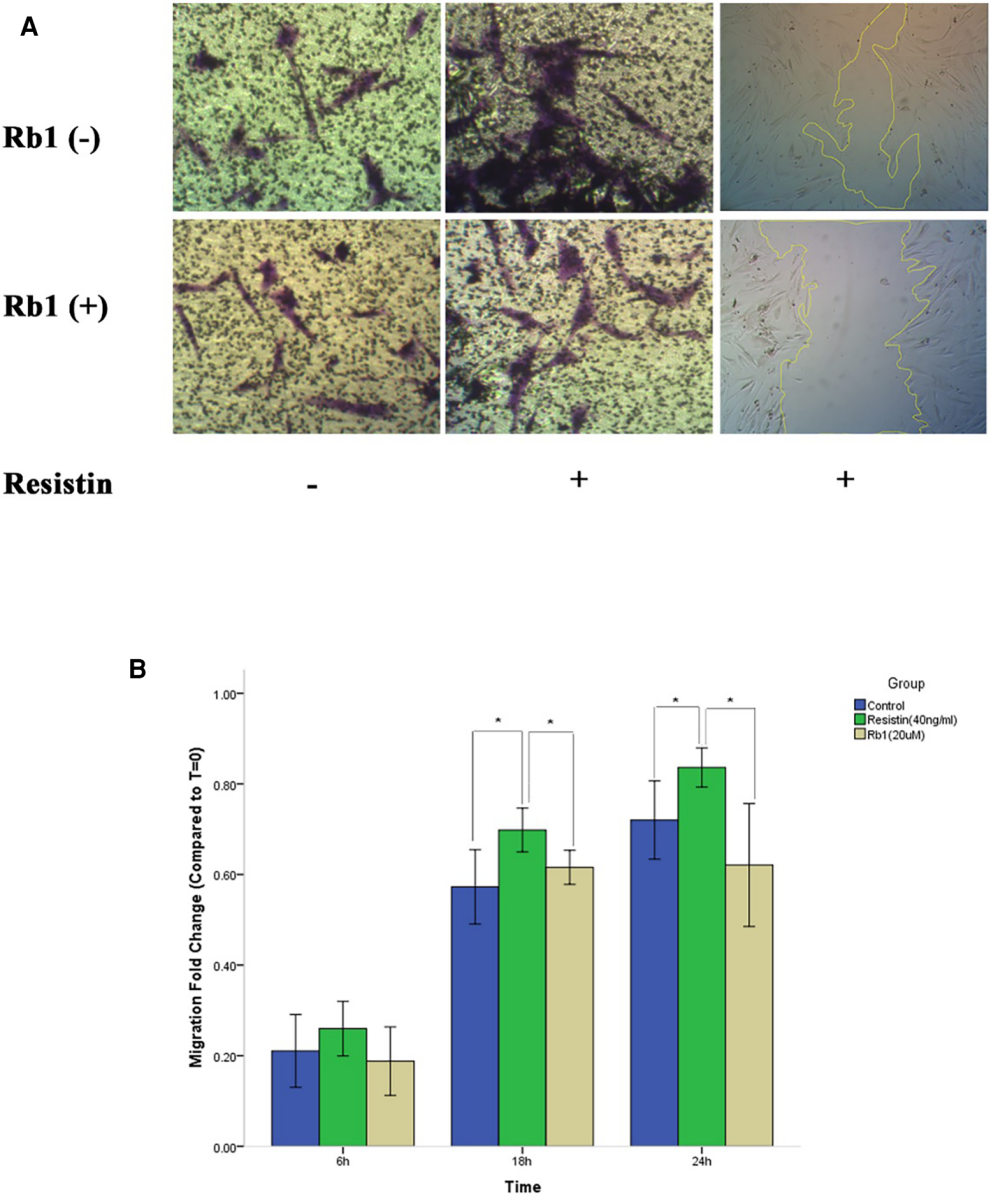
Rb1 inhibited resistin-induced HCASMC migration. (**A**) Transwell migration chamber assay and wound healing assay showed resistin-induced HCASMC migration was inhibited by Rb1. (**B**) Cell migration was calculated as the fold change of wound closure relative to controls (*T* = 0). Resistin (40 ng/ml) treatment time dependently increased HCASMC migration, which could be significantly attenuated by Rb1 (20 µM) at 18 h and 24 h (**B**). Values were means and SD (*n* = 5). * *P* < 0.05.

To elucidate the effects of Rb1 on HCASMC migration, a wound healing assay was used. The wound healing assay revealed resistin at 40 ng/ml treatment time dependently increased VSMCs migration, which was attenuated significantly by Rb1 at 20 µM at 18 h and 24 h (Figure 2). A transwell migration chamber assay was also used to further identify the chemotaxis effect of resistin with or without Rb1. As shown in Figure 2, resistin increased the migratory ability of HCASMCs, whereas Rb1 inhibited the resistin-induced HCASMC migration.

### Effect of Rb1 on intracellular ROS production of HCASMC

**Figure 3:**
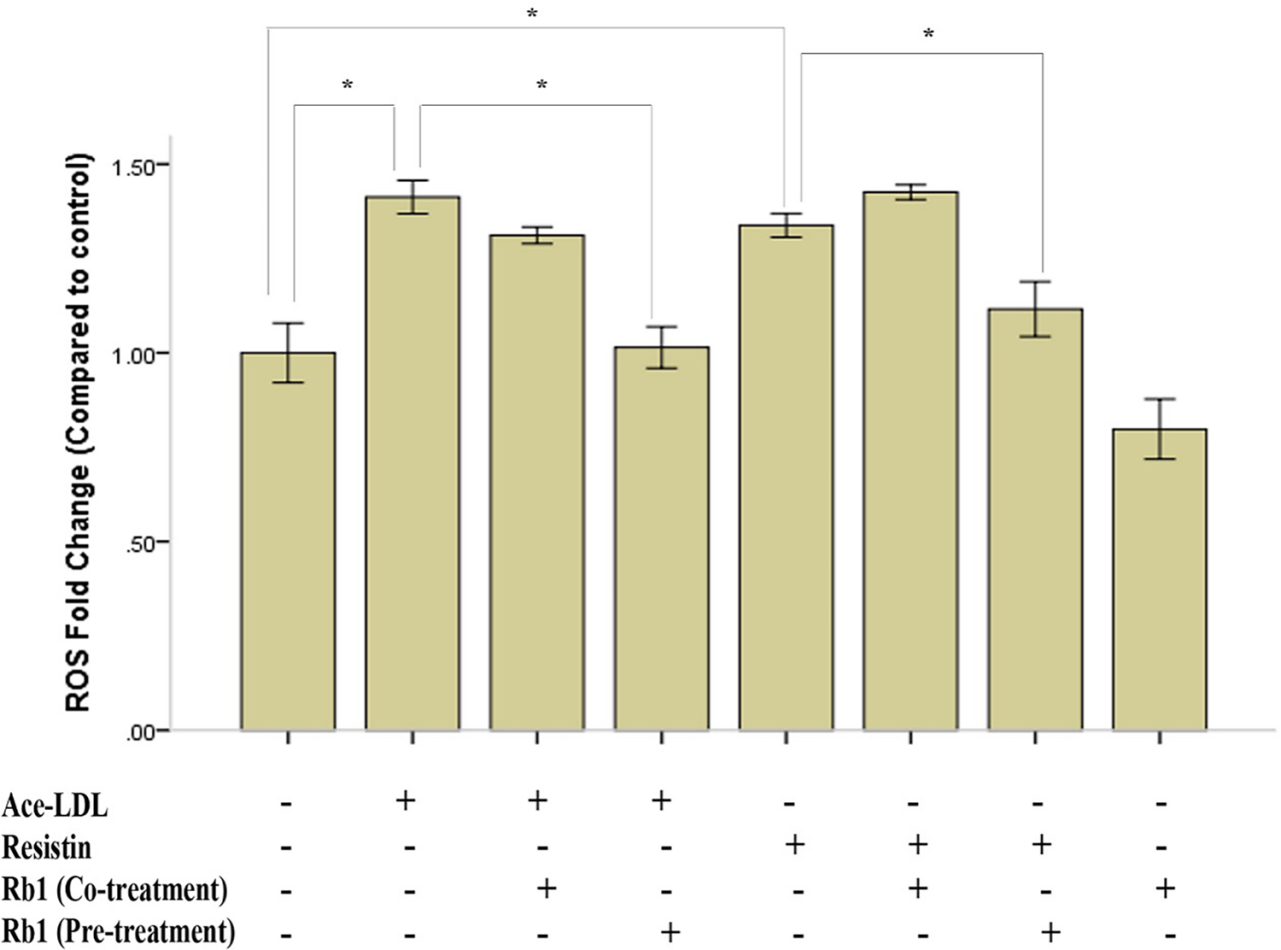
Rb1 pretreatment reduced resistin-induced ROS production in HCASMC. HCASMC ROS production was detected by DCF-DA fluorescence assay. After treated with resistin (40 ng/ml) or acetyl-LDL (20 µg/ml) for 2 h, the intracellular ROS were significantly increased to a similar level. Rb1 pretreatment (24 h) significantly reduced HCASMC ROS generation induced by resistin and acetyl-LDL, while Rb1 co-treatment (2 h) had no such effect. Values were means and SD (*n* = 5). *P < 0.05.

**Figure 4:**
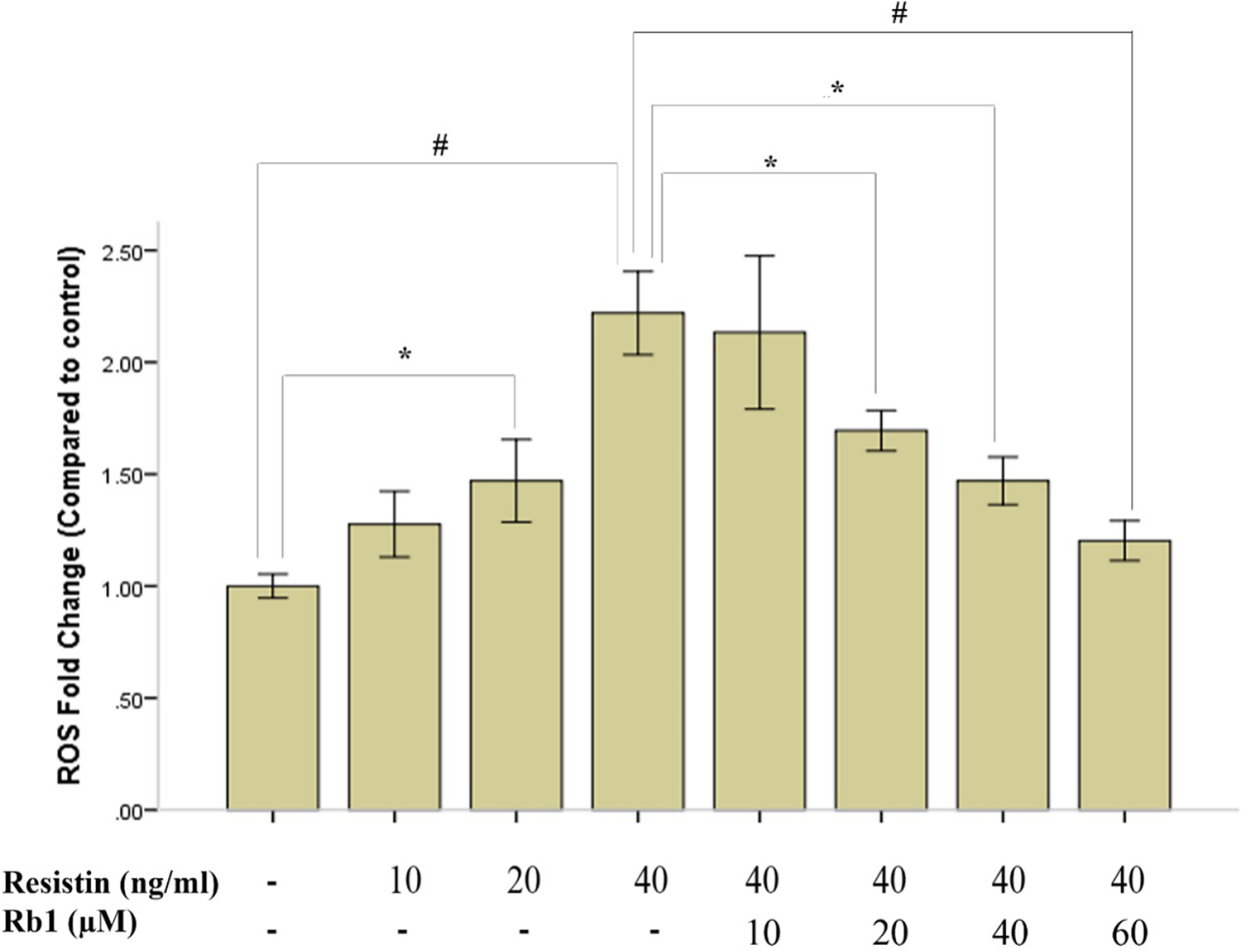
Rb1 pretreatment dose dependently inhibited resistin-induced HCASMC ROS generation. Resistin dose dependently increased ROS generation in HCASMC, which could be significantly inhibited by Rb1 (>20 µM) in a dose-dependent manner. Values were means and SD (*n* = 5). *P < 0.05, #*P* < 0.01.

It has been well-documented that high expressed ROS could lead to destructive impact on both differentiated EC and VSMC (21). Since ROS generations are key instigators in the pathophysiology of resistin-associated VSMC dysfunction and intimal hyperplasia (14), we determined whether Rb1 was able to inhibit resistin-induced ROS production in HCASMC by DCF-DA fluorescence assay. We found that both resistin and acetyl-LDL could increase ROS production in HCASMC to a similar level, and while pretreated with Rb1, ROS production induced by resistin and acetyl-LDL were reduced significantly (Figure 3). In addition, we proved that both Rb1 and resistin have dose-dependent effects on ROS production in HCASMC, suggesting Rb1 at a concentration of 20 µM could significantly reduce resistin (40 ng/ml) induced cytosolic ROS in VSMCs (Figure 4). It was interesting to find that Rb1 pretreatment was more effective than co-treatment in restoring ROS levels induced by resistin in HCASMC (Figure 3).

### Rb1 attenuated resistin-induced suppression of mitochondrial SOD activity in HCASMC

**Figure 5:**
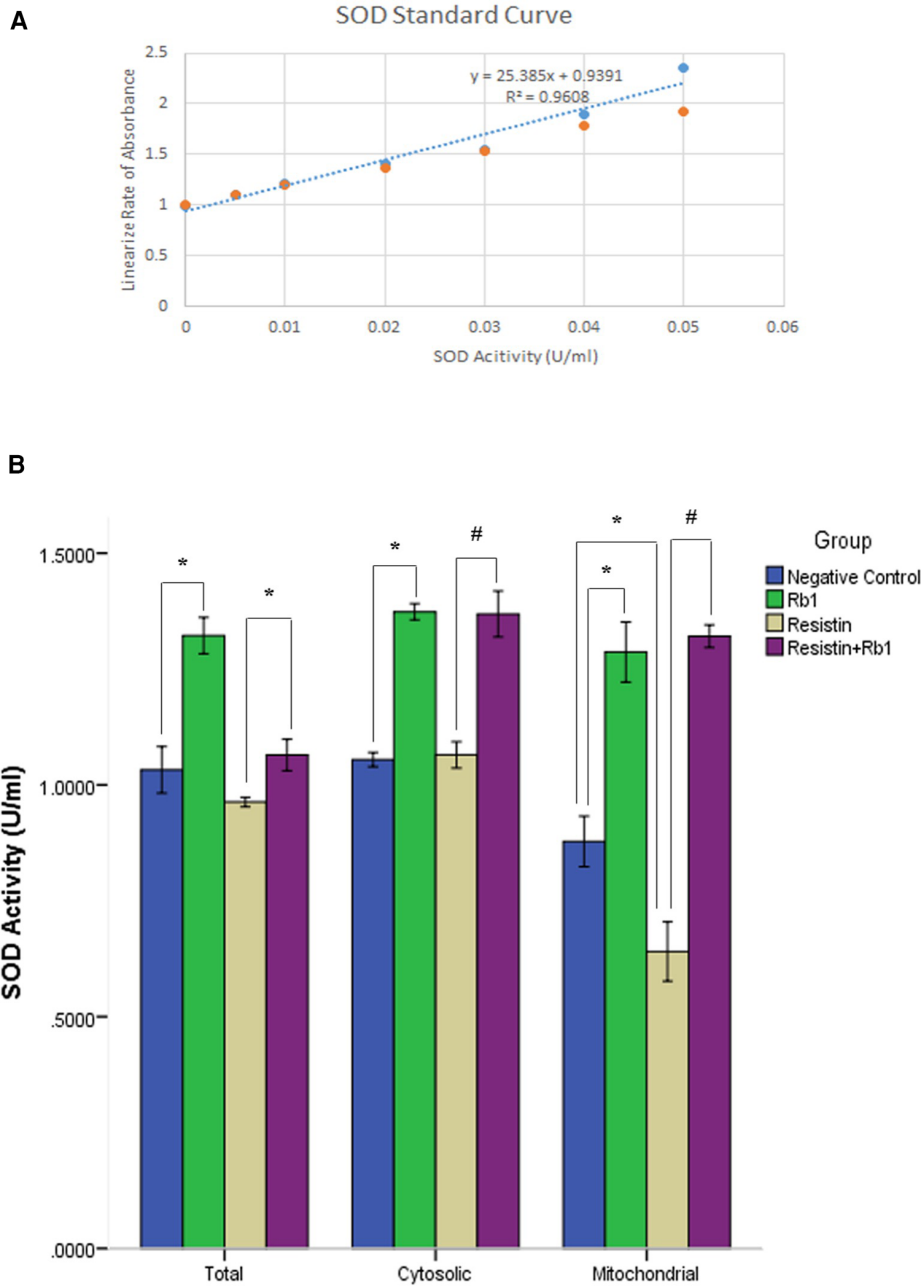
Rb1 attenuated resistin-induced suppression of HCASMC SOD activity. (**A**) The SOD activity of the samples was calculated from the linear regression of the standard curve. One unit of SOD is defined as the amount of enzyme needed to exhibit 50% dismutation of superoxide radical. (**B**) The mitochondrial SOD activity was significantly reduced by resistin. In contrast, Rb1 significantly increased both cytosolic and mitochondrial SOD activity in HCASMC, which attenuated resistin-induced reduction of mitochondrial SOD activity. Values were means and SD (*n* = 5). *P < 0.05, #*P* < 0.01.

In order to clarify the pathogenesis of the antioxidation of Rb1 in HCASMC exposed to resistin, the catalytic activity of SOD was analyzed by the measurement of dismutation of superoxide radical in HCASMC that had been treated with Rb1 for 24 h followed by 40 ng/ml of resistin for additional 2 h. Resistin reduced the total SOD activity in HCASMC which was shown in Figure 5; however, pretreatment with Rb1 (20 µM) significantly attenuated the resistin-induced suppression of the total SOD activity. Rb1 alone was capable to exert its antioxidation by increasing the SOD activity in HCASMC. To further target the specific part of HCASMC on which resistin and Rb1 took effect, we analyzed the cytosolic and mitochondrial SOD activity within HCASMC. Our data revealed that the mitochondrial SOD activity was significantly reduced by resistin but was significantly increased when pretreated by Rb1 (*P* < 0.01). This suggested resistin might increase ROS production of HCASMC partially through reducing the mitochondrial SOD activity, which could be significantly reversed by Rb1.

## Discussion

Dysfunction of VSMCs and macrophages are the main causes of atherosclerotic plaque progression. Recent studies suggest that the crosstalk between VSMC and monocytes/ macrophages represents a great role for the atherosclerosis and leads to up-regulation of resistin in monocytes (18, 22). Resistin is a type of cytokine produced by monocytes/macrophages in human atheroma, and a grow number of clinical evidence indicates that resistin may independently predict major adverse cardiovascular events (18, 23). Additionally, resistin has been demonstrated to play a critical role in inflammation and intracellular ROS production of VSMCS that are closely relevant in the pathogenesis of cardiovascular disease (17, 18). However, the relevant mechanism and new therapeutic strategies remain uncertain. Our study represents a new potential treatment option by demonstrating that resistin and acetyl-LDL induced HCASMCs migration and proliferation could be significantly attenuated by Rb1, which was related to the reduction of ROS and the increase of SOD activity in HCASMCs. Our data suggested ginsenoside Rb1 has the potential of protecting against resistin-induced pathophysiological changes of atherosclerosis.

Recently, the positive effects of ginsenosides on CVD were confirmed including their ability of controlling ROS, production of nitric oxide (NO), and the activation of various receptors in endothelial cells (24). We first reported ginsenoside Rb1 could cut off superoxide anion production, endothelial dysfunction and eNOS down-regulation of porcine coronary arteries by homocysteine (5). Other studies also suggested that Rb1 protected human aortic and umbilical vein endothelial cells of human through the production and release of nitric oxide in the endothelium *via* PI3K/Akt activation and PKC inhibition (25, 26). Zhang et al. proved that Rb1 restricted the inflammatory response and promoted M2 macrophage polarization, which further helps to stabilize atherosclerosis progression in ApoE^−/−^ mice (4). These reports, however, are limited mostly to data of endothelial cells, with few studies examining the protective effect of Rb1 on VSMCs. Li et al. found Rb1 could inhibit TNF-α-evoked inflammatory responses and FBS-induced proliferation *in vitro* experiment of rats (28). In a rat model, Zhang demonstrated that Rb1 could inhibited neointimal hyperplasia after balloon dilation by inhibition of VSMC proliferation (8). However, the aforementioned studies are confined to the level of rat VSMCs. In the present study, the protective effect of Rb1 on HCASMCs dysfunction induced by resistin and acetyl-LDL was demonstrated by wound healing assay and MTS assay. As far as we know, this is the first study to evaluate the role of Rb1 in human VSMC dysfunction induced by resistin.

It is worth noting that the protective effect of Rb1 was related to the ROS reduction within VSMCs as shown in our study. ROS are key signaling molecules that regulate vascular function and structure under physiological conditions. However, excessive ROS expression may be associated with the progression of several adverse cardiovascular diseases, such as VSMC proliferation and vascular remodeling caused by inflammatory reaction (21). At the intracellular level, the activation of mitogen activated protein kinases (MAPKs) in ROS-dependent signaling pathways in VSMCs involves p38, extracellular signal-regulated kinases (ERK1/2), c-Jun N-terminal kinases and the serine/threonine kinase Akt/protein kinase, which play a critical role in cell proliferation and migration and pathological remodeling (21, 28). We previously have proved that resistin-related intimal hyperplasia and VSMC dysfunction were associated with ROS generation and PKC*ε*-dependent Nox activation (17). In the present study, we further demonstrated resistin-induced ROS production, which was partially through suppression of SOD activity, could be reversed by Rb1 pretreatment. Interestingly, we found that Rb1 pretreatment was more effective in reversing this disorder than co-treatment. In the co-treatment group of ROS experiment, the exposure time of 2 h was not long enough for Rb1 to take effect on VSMCs but was sufficient for resistin to generate a large amount of ROS, which resulted in the dysfunction of VSMCs afterwards. This suggested Rb1 requires a relatively long period of time (more than 2 h) to exert its antioxidative effect on VSMCs.

Besides NADPH oxidase, it is generally believed that ROS in living cells including vascular cells mainly comes from mitochondria (29). In the present study, we observed that resistin significantly triggered ROS generation by targeting the mitochondrion SOD activity and causing an imbalance of oxidative stress within VSMCs, which could be reversed by Rb1. The mitochondrion SOD is comprised of three isoforms: cytosolic Cu/Zn-SOD (SOD_1_), mitochondrial Mn-SOD (SOD_2_) and extracellular EC-SOD (SOD_3_) (21). The regulation of Rb1 on mitochondrial ROS involves the regulation of oxidase and antioxidant enzymes to inhibit the excessive production and accumulation of ROS. As previously mentioned, after treatment with Rb1, through activation of the PI3K/Akt/Nrf2 signaling pathway, serum or tissue level of SOD were increased in spinal cord injury and intestinal ischemia/reperfusion model rats (30–32). In vitro study, Rb1 could markedly increase intracellular SOD activity in ritonavir-treated human endothelial cells and inhibit the production of intracellular ROS (34). Thus, in consistent with this, our results showed Rb1 effectively exerted antioxidation by regulating SOD activity to relieve resistin-induced mitochondrial damage in VSMCs.

There are some limitations in our study. Although we confirmed the protection of Rb1 on VSMCs was related to the increase of SOD activity, the dynamic equilibrium of ROS production and elimination is far more complicated and needs to be further elucidated. For example, the elimination of superoxide anions is the consequence of coaction of several antioxidant enzymes. SOD catalyzes the dismutation of superoxide anions into H_2_O_2_ and oxygen, and H_2_O_2_ can be metabolized rapidly to water and oxygen by enzyme-linked reactions (34, 35). Whether Rb1 has a positive effect on other antioxidant enzymes, such as catalase, remains to be investigated. Secondly, the exact molecular mechanism for the protective effect of Rb1 has not been fully revealed in the present study. Numerous studies have identified that Rb1 exhibits antioxidant effects by activating the PI3K/Akt pathway with subsequent Nrf2 nuclear translocation and induction of antioxidant enzymes (30–32, 36–38). Since we have shown the protective effect of Rb1 was related to the antioxidation of SOD, which is one of the downstream productions of Nrf2 gene, we speculated that the part of the mechanism underlying the beneficial effects of Rb1 on resistin-induced VSMC dysfunction might be related to ROS dependent PI3K/Akt/Nrf2 signaling pathway. However, further studies will be required to examine this hypothesis.

## Conclusion

This study provides new evidence supporting the hypothesis that ginsenoside Rb1can inhibit resistin-induced proliferation and migration in HCASMCs. Furthermore, we showed that the mechanisms involved in these effects exerted by Rb1 might be related to the reduction of ROS generation and increased activity of SOD. However, the molecular mechanism for the protective effect of Rb1 is far more complicated and needs to be further elucidated. The findings presented here also highlight the potential clinical applications of Rb1 in controlling resistin-associated vascular injury and the possible therapeutic use in cardiovascular disease.

## Data Availability

The original contributions presented in the study are included in the article/**Supplementary Material**, further inquiries can be directed to the corresponding authors.
