# Supplementary material for: Ginsenoside Rb1 protects human vascular smooth muscle cells against resistin-induced oxidative stress and dysfunction
Source: Front Cardiovasc Med. 2023 May 25;10:1164547. doi: 10.3389/fcvm.2023.1164547 (PMC10248054; doi:10.3389/fcvm.2023.1164547)
Supplement: Supplementary file 1 [file Datasheet1.zip › Raw data/Fig 3 ROS/Lab meeting 20190124.pptx]

## Slide 1
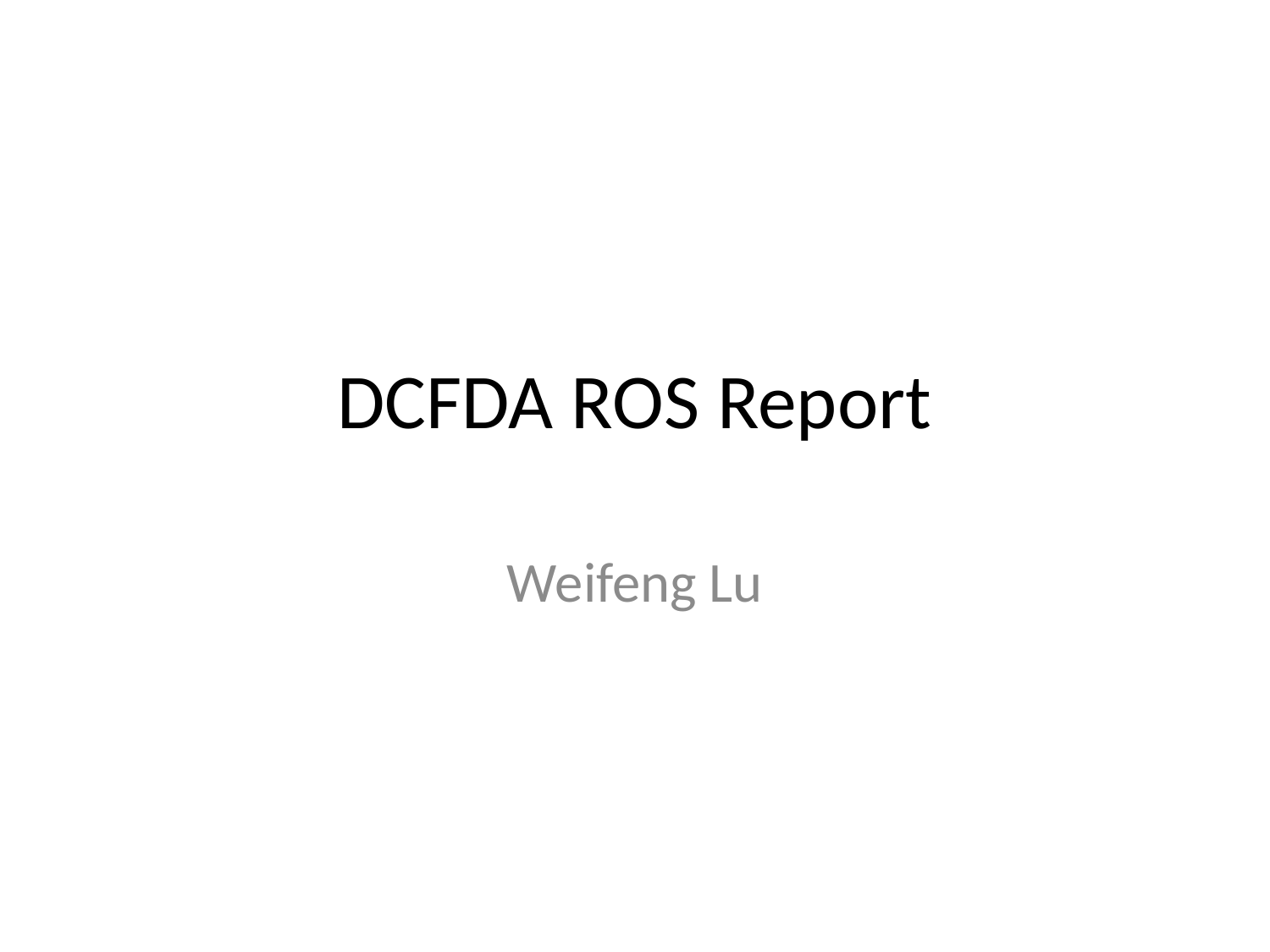

# DCFDA ROS Report
Weifeng Lu

## Slide 2
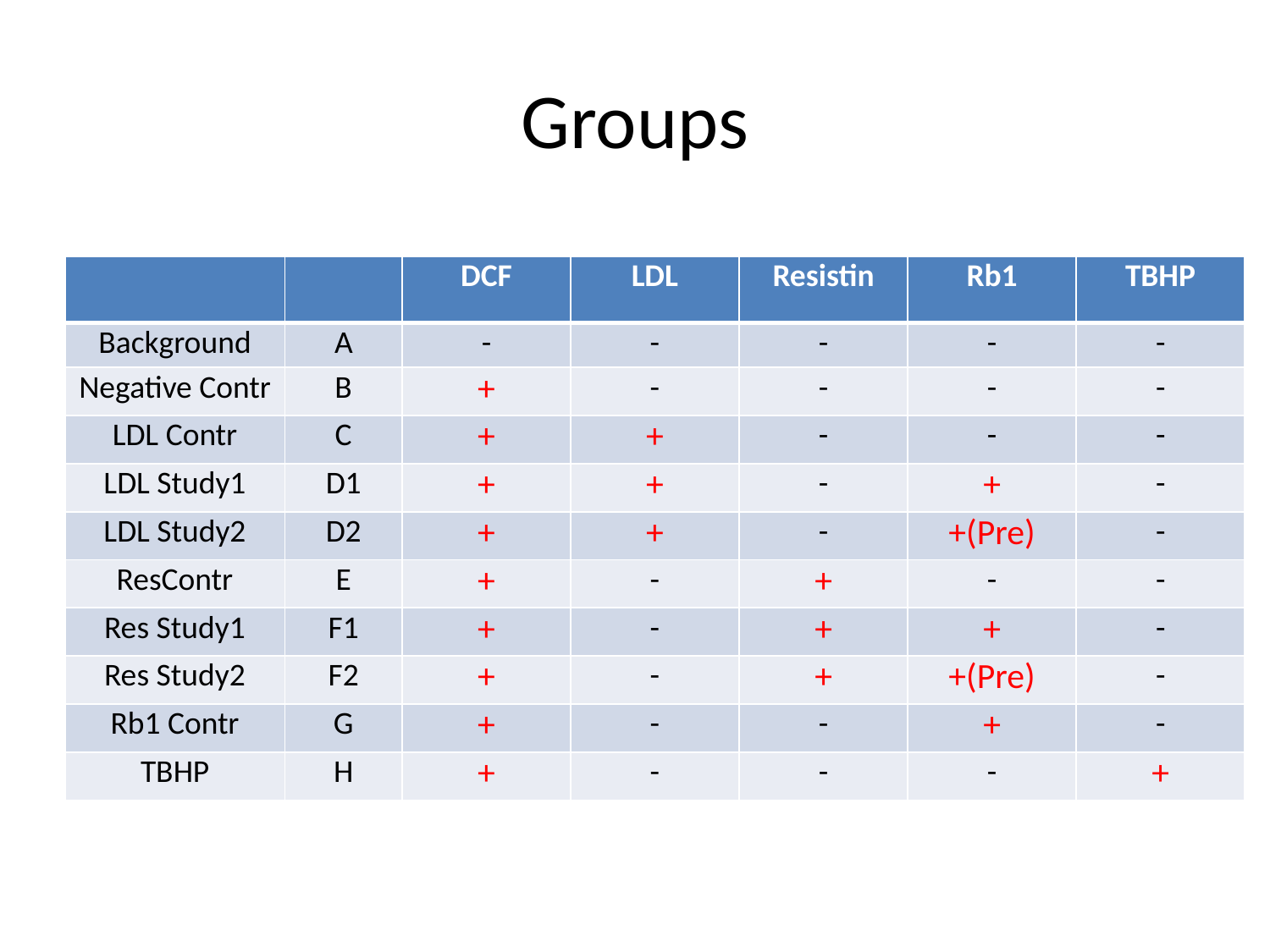

# Groups
| | | DCF | LDL | Resistin | Rb1 | TBHP |
| --- | --- | --- | --- | --- | --- | --- |
| Background | A | - | - | - | - | - |
| Negative Contr | B | + | - | - | - | - |
| LDL Contr | C | + | + | - | - | - |
| LDL Study1 | D1 | + | + | - | + | - |
| LDL Study2 | D2 | + | + | - | +(Pre) | - |
| ResContr | E | + | - | + | - | - |
| Res Study1 | F1 | + | - | + | + | - |
| Res Study2 | F2 | + | - | + | +(Pre) | - |
| Rb1 Contr | G | + | - | - | + | - |
| TBHP | H | + | - | - | - | + |

## Slide 3
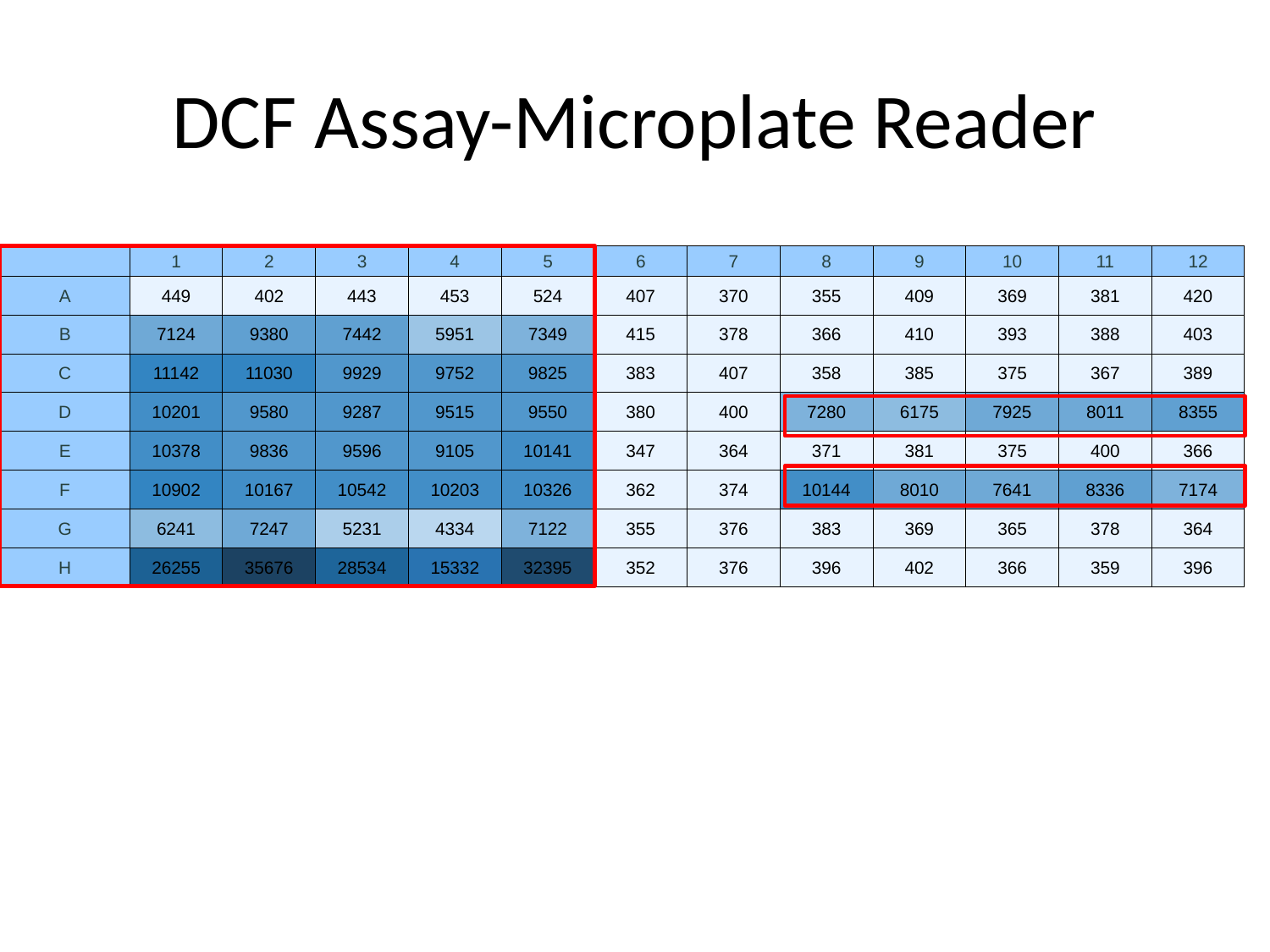

# DCF Assay-Microplate Reader
| | 1 | 2 | 3 | 4 | 5 | 6 | 7 | 8 | 9 | 10 | 11 | 12 |
| --- | --- | --- | --- | --- | --- | --- | --- | --- | --- | --- | --- | --- |
| A | 449 | 402 | 443 | 453 | 524 | 407 | 370 | 355 | 409 | 369 | 381 | 420 |
| B | 7124 | 9380 | 7442 | 5951 | 7349 | 415 | 378 | 366 | 410 | 393 | 388 | 403 |
| C | 11142 | 11030 | 9929 | 9752 | 9825 | 383 | 407 | 358 | 385 | 375 | 367 | 389 |
| D | 10201 | 9580 | 9287 | 9515 | 9550 | 380 | 400 | 7280 | 6175 | 7925 | 8011 | 8355 |
| E | 10378 | 9836 | 9596 | 9105 | 10141 | 347 | 364 | 371 | 381 | 375 | 400 | 366 |
| F | 10902 | 10167 | 10542 | 10203 | 10326 | 362 | 374 | 10144 | 8010 | 7641 | 8336 | 7174 |
| G | 6241 | 7247 | 5231 | 4334 | 7122 | 355 | 376 | 383 | 369 | 365 | 378 | 364 |
| H | 26255 | 35676 | 28534 | 15332 | 32395 | 352 | 376 | 396 | 402 | 366 | 359 | 396 |

## Slide 4
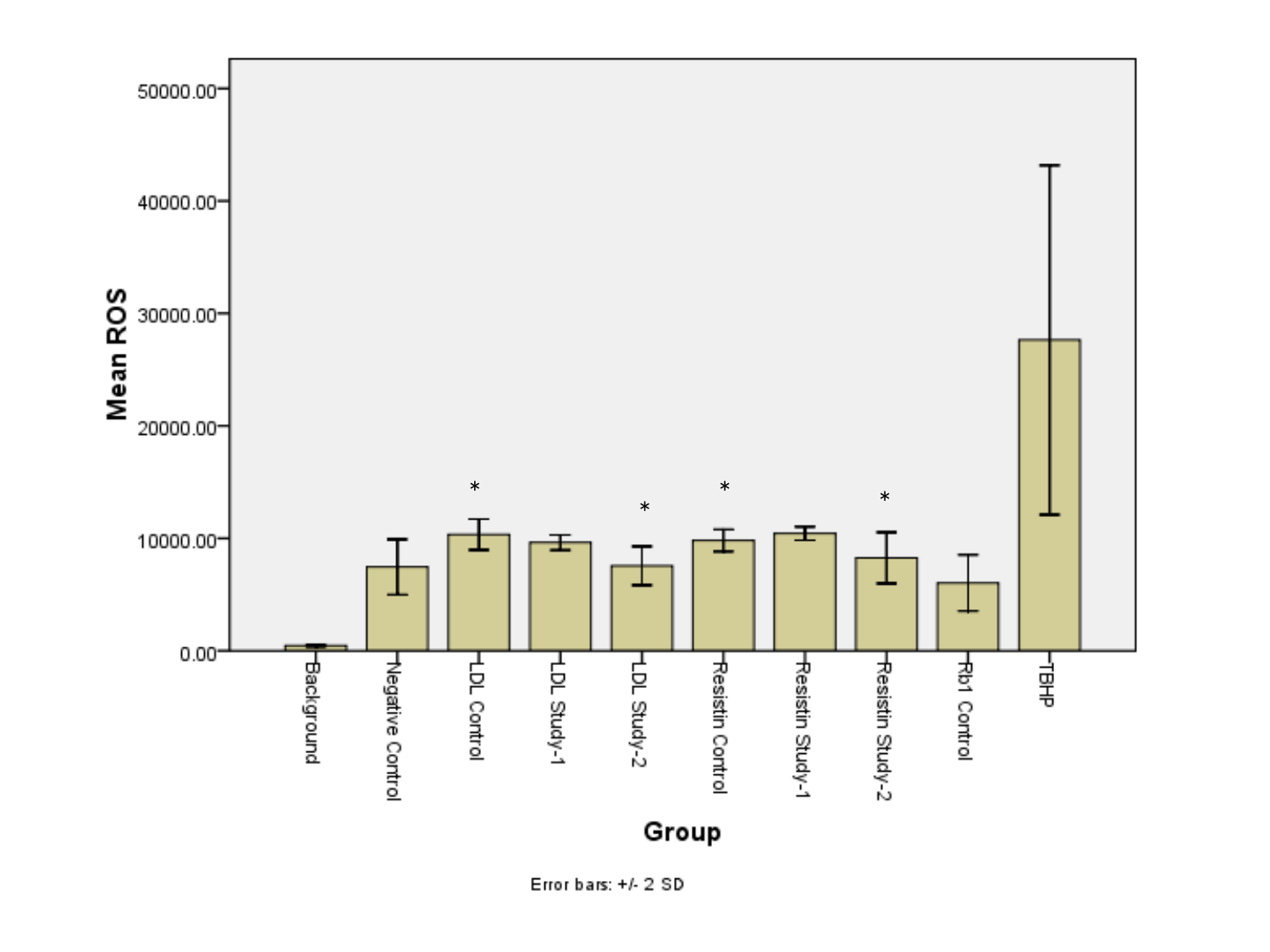

#
*
*
*
*

## Slide 5
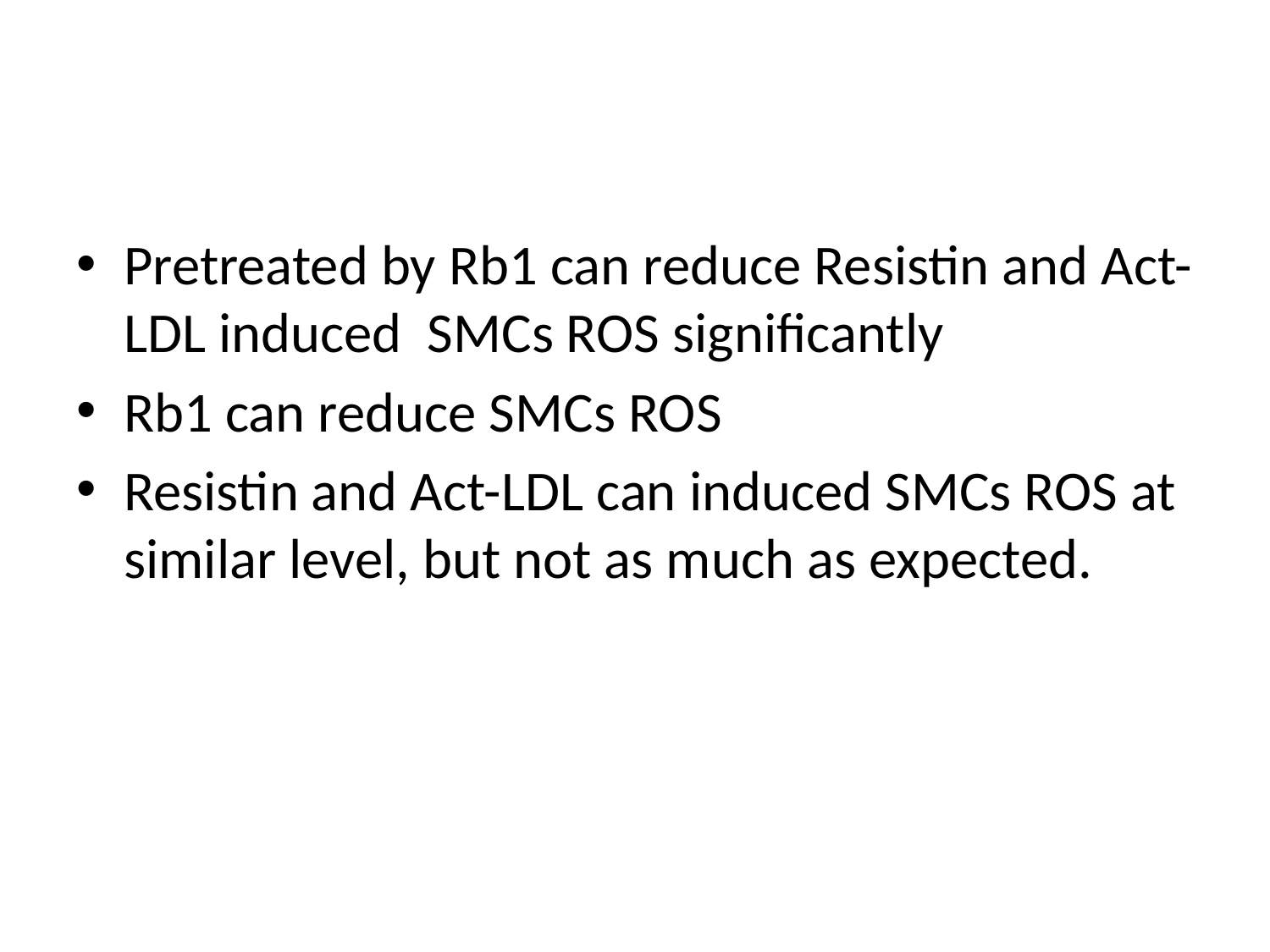

#
Pretreated by Rb1 can reduce Resistin and Act-LDL induced SMCs ROS significantly
Rb1 can reduce SMCs ROS
Resistin and Act-LDL can induced SMCs ROS at similar level, but not as much as expected.

## Slide 6
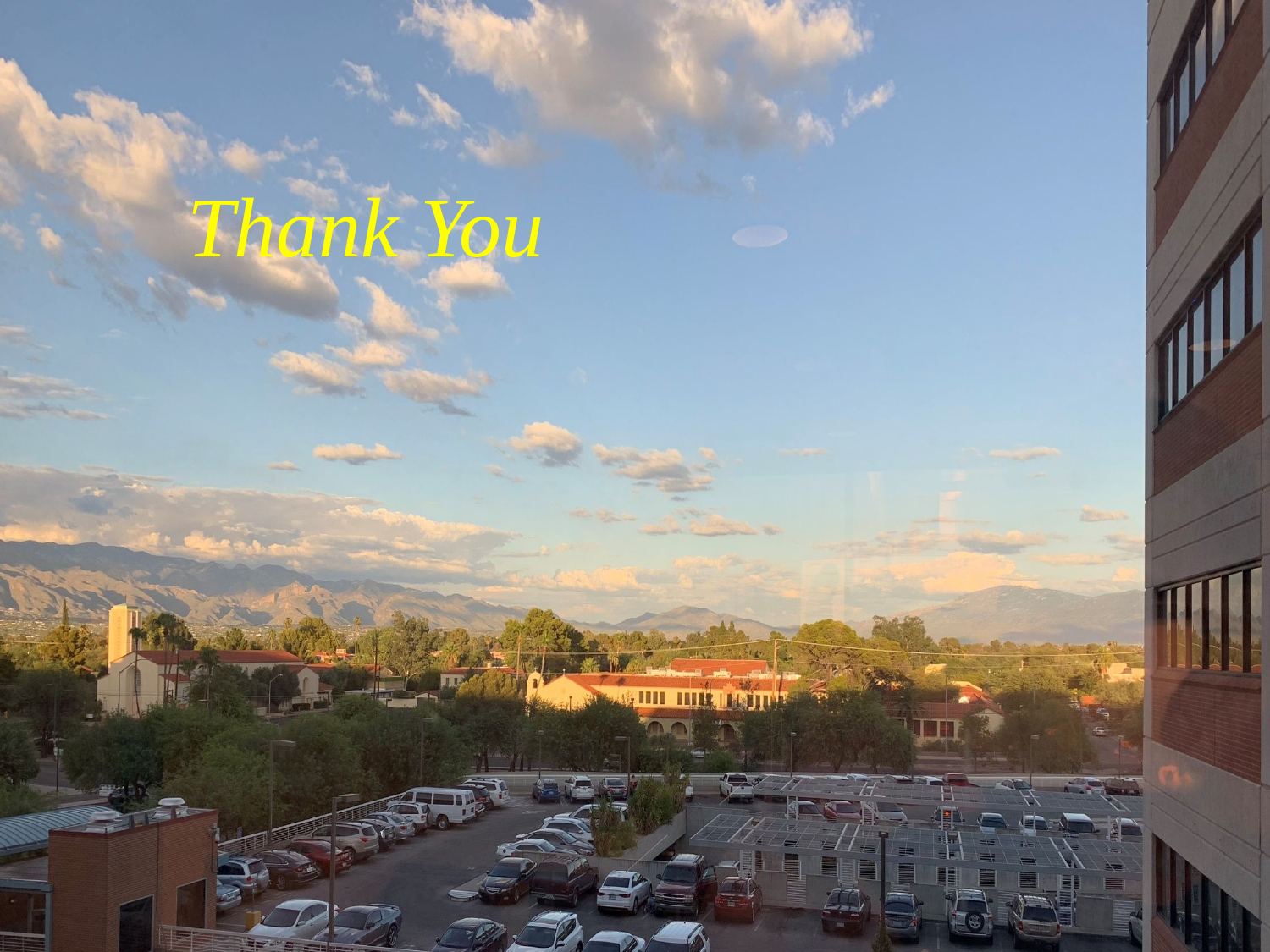

#
Thank You

## Slide 7
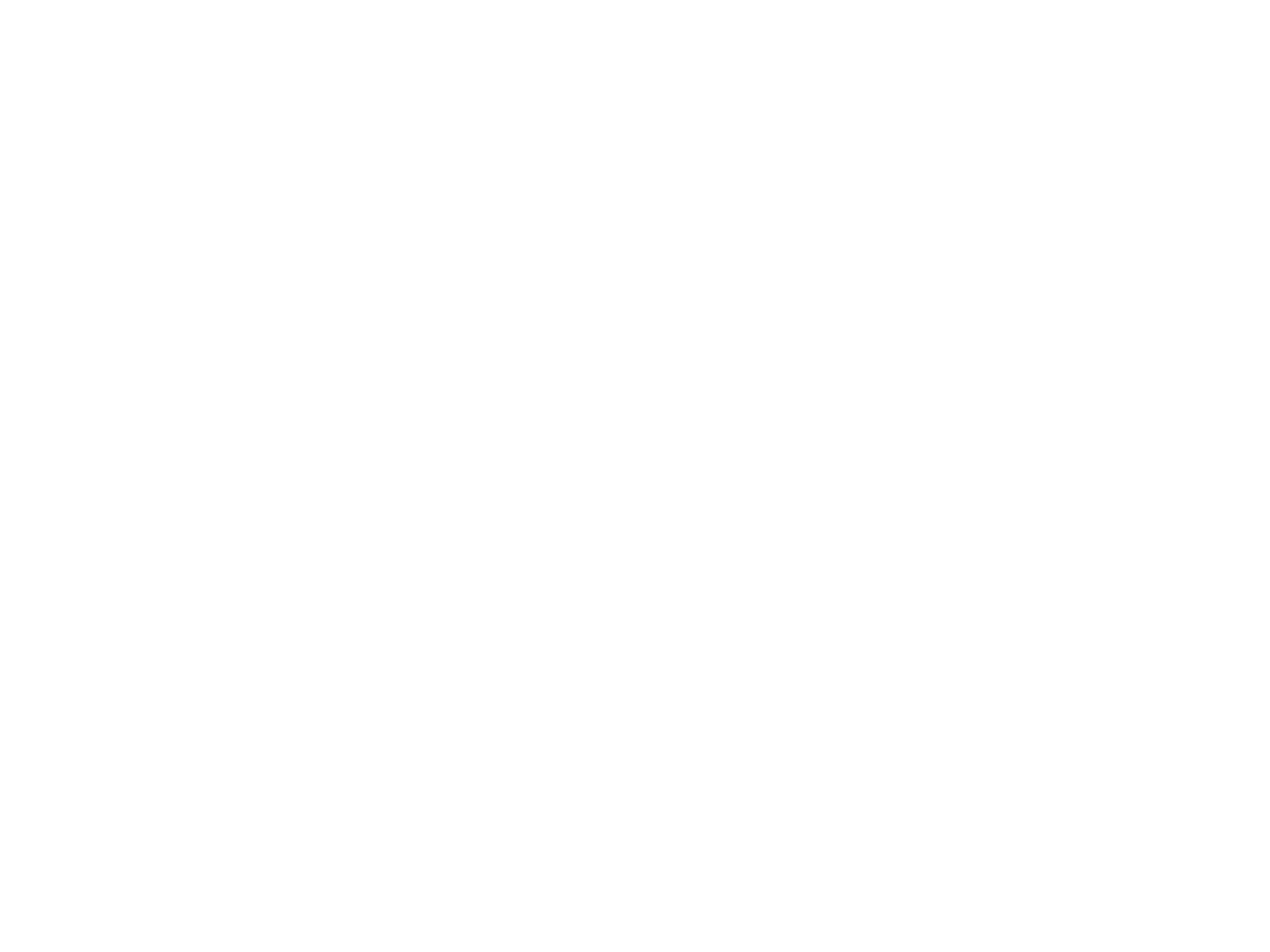

#

## Slide 8
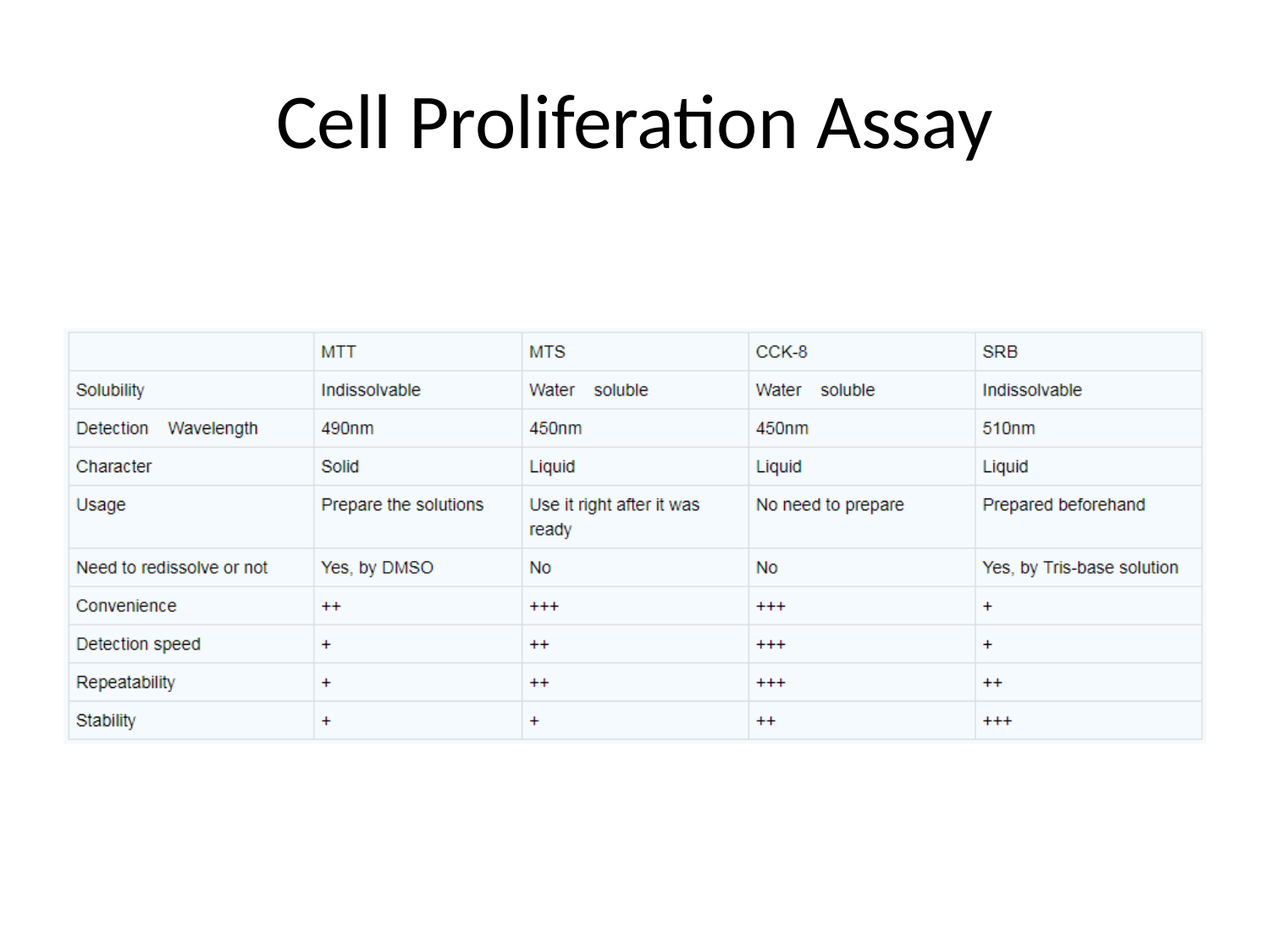

# Cell Proliferation Assay

## Slide 9
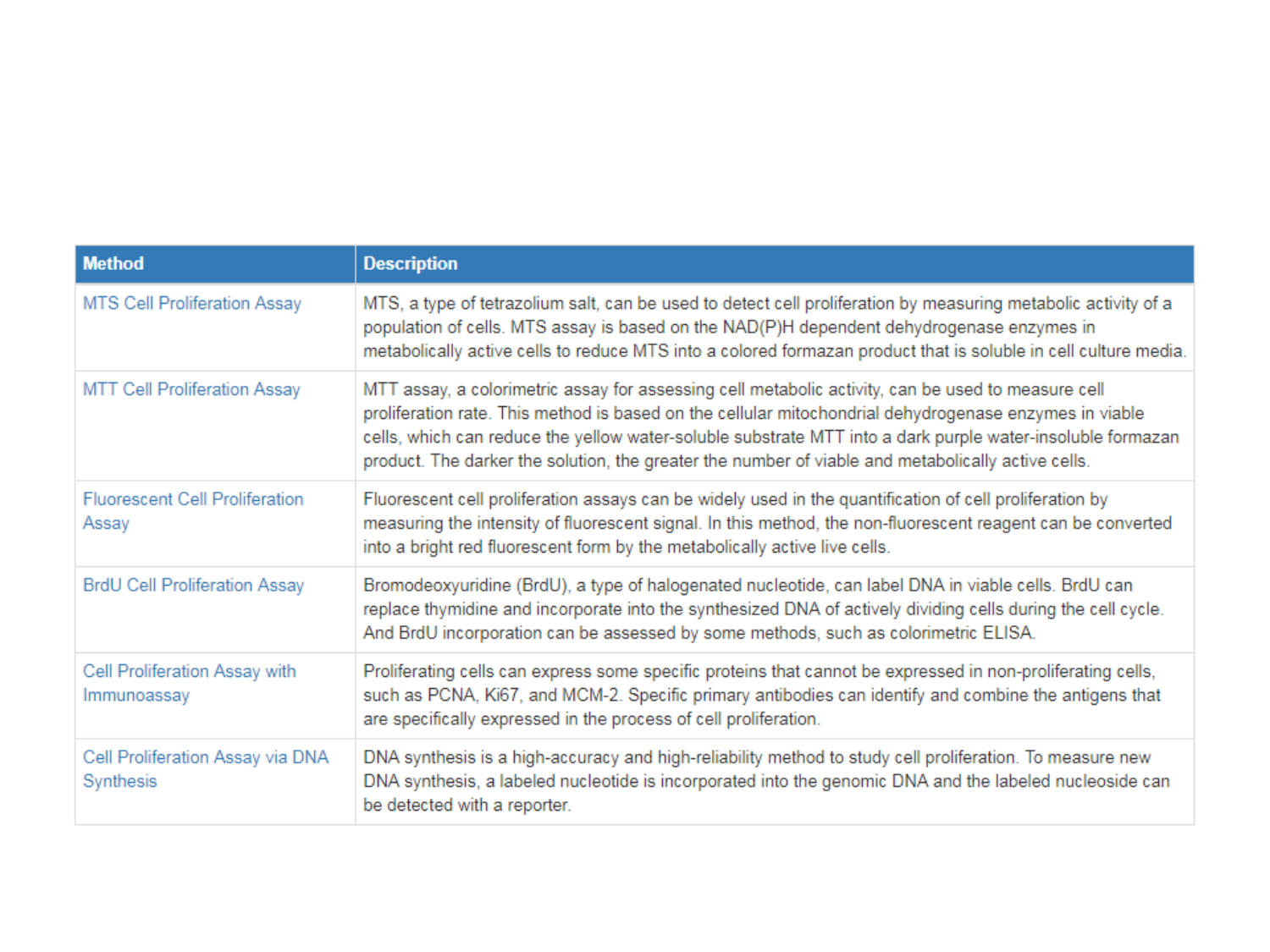

#
