# Supplementary material for: Ginsenoside Rb1 protects human vascular smooth muscle cells against resistin-induced oxidative stress and dysfunction
Source: Front Cardiovasc Med. 2023 May 25;10:1164547. doi: 10.3389/fcvm.2023.1164547 (PMC10248054; doi:10.3389/fcvm.2023.1164547)
Supplement: Supplementary file 1 [file Datasheet1.zip › Raw data/Fig 1 MTS/Lab meeting 20190408.pptx]

## Slide 1
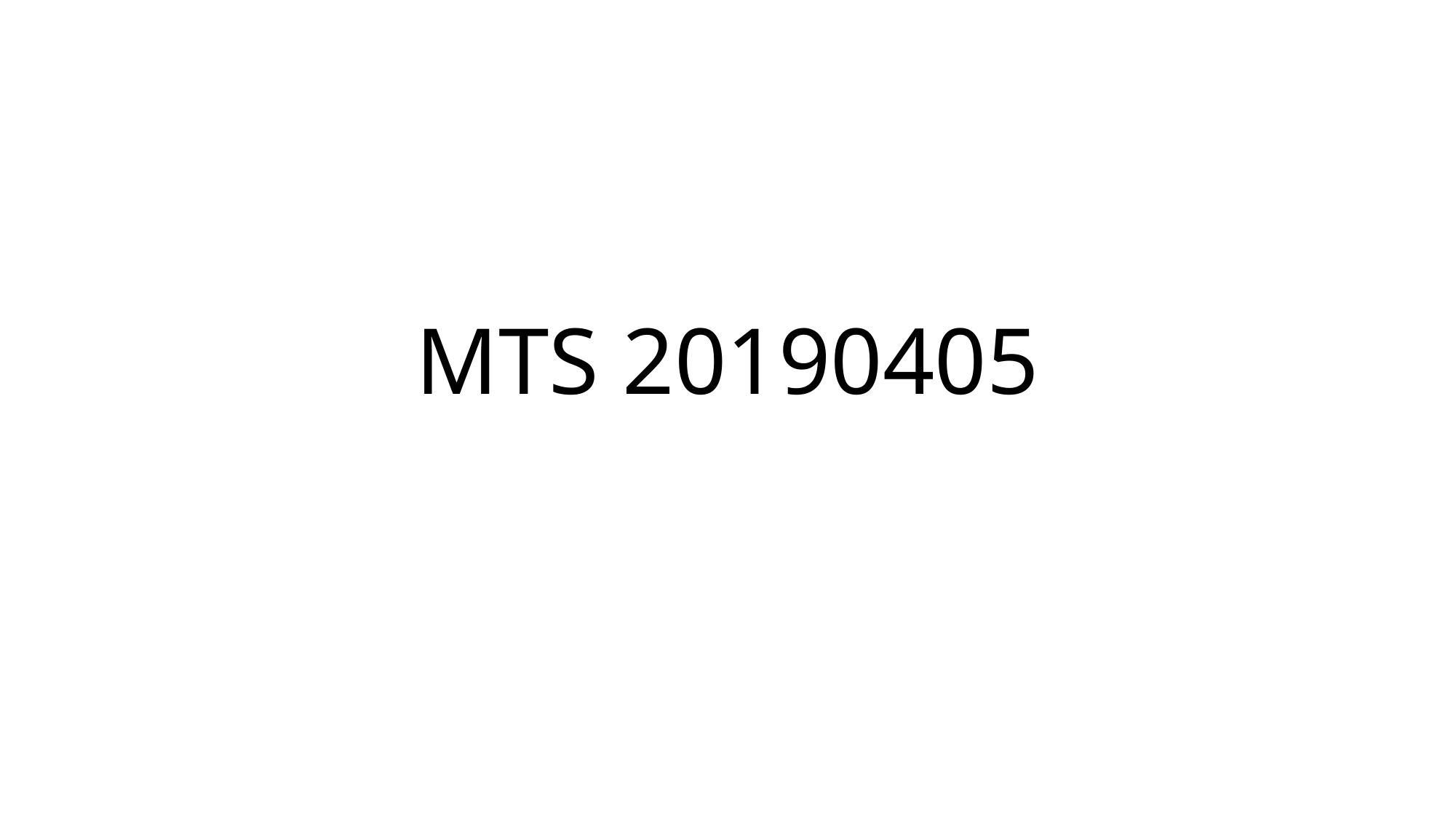

# MTS 20190405

## Slide 2
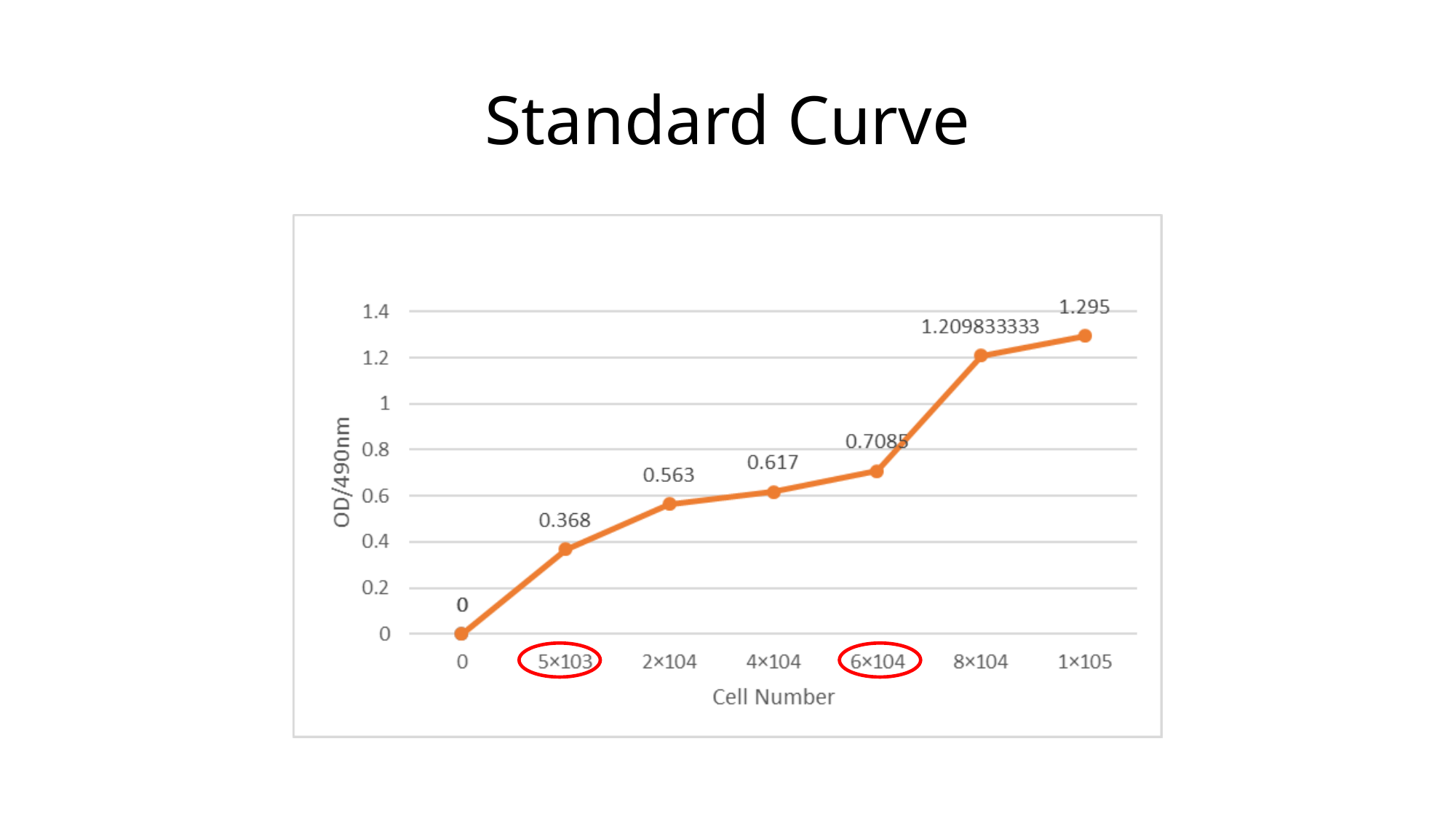

# Standard Curve

## Slide 3
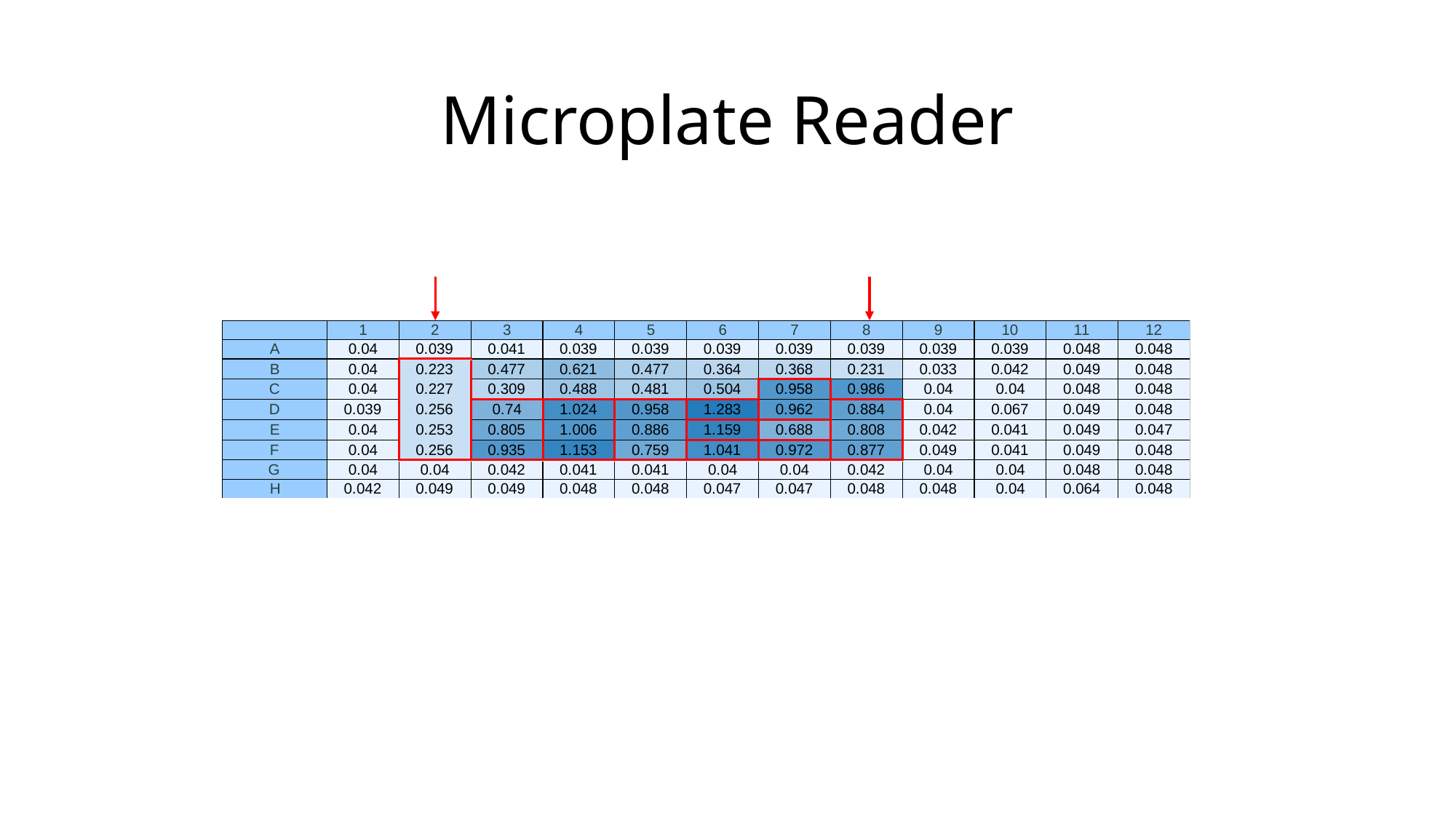

# Microplate Reader

## Slide 4
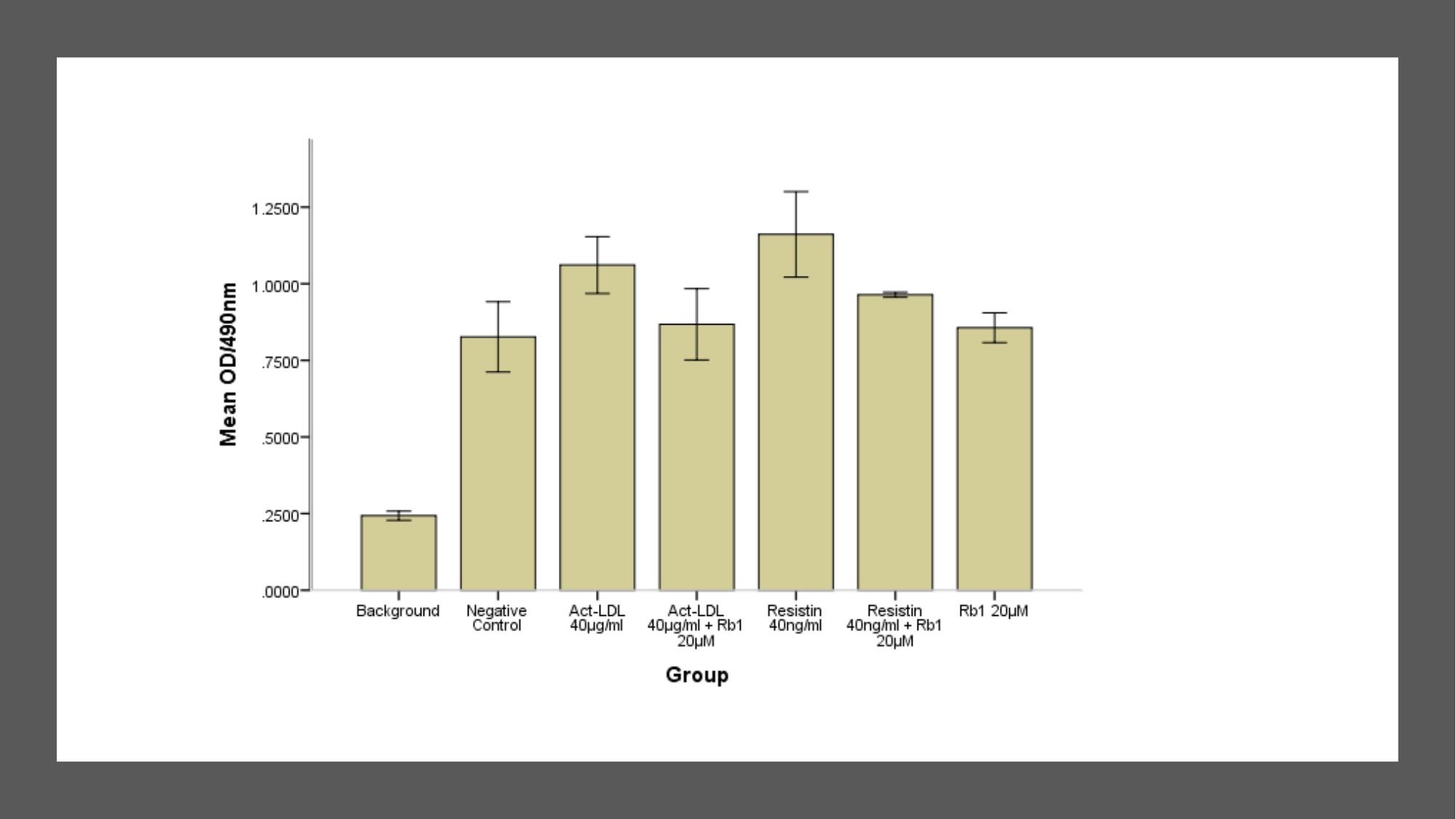

## Slide 5
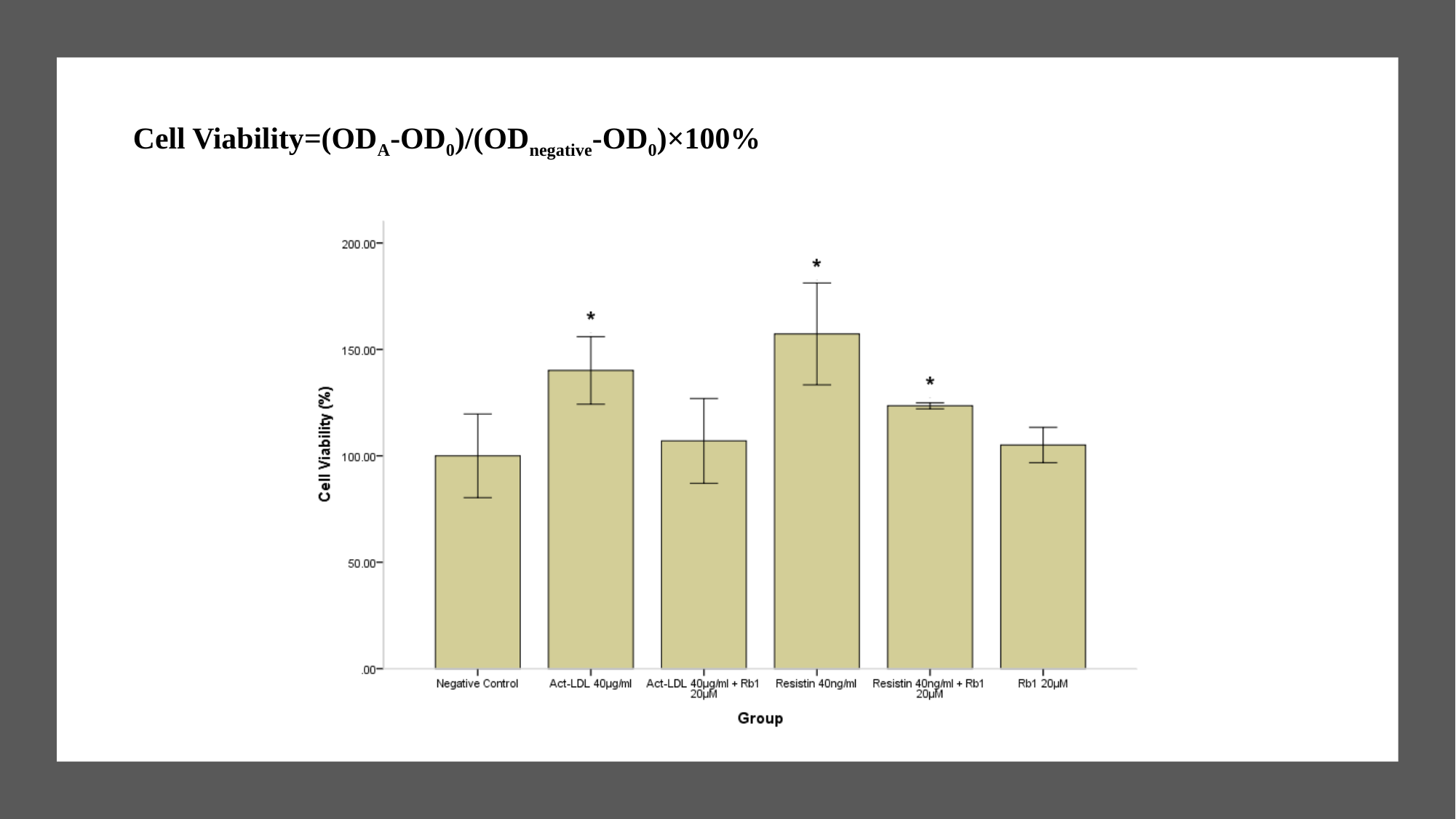

Cell Viability=(ODA-OD0)/(ODnegative-OD0)×100%
