# Supplementary material for: Ginsenoside Rb1 protects human vascular smooth muscle cells against resistin-induced oxidative stress and dysfunction
Source: Front Cardiovasc Med. 2023 May 25;10:1164547. doi: 10.3389/fcvm.2023.1164547 (PMC10248054; doi:10.3389/fcvm.2023.1164547)
Supplement: Supplementary file 1 [file Datasheet1.zip › Raw data/Fig 2 Migration/Lab meeting 20181210.pptx]

## Slide 1
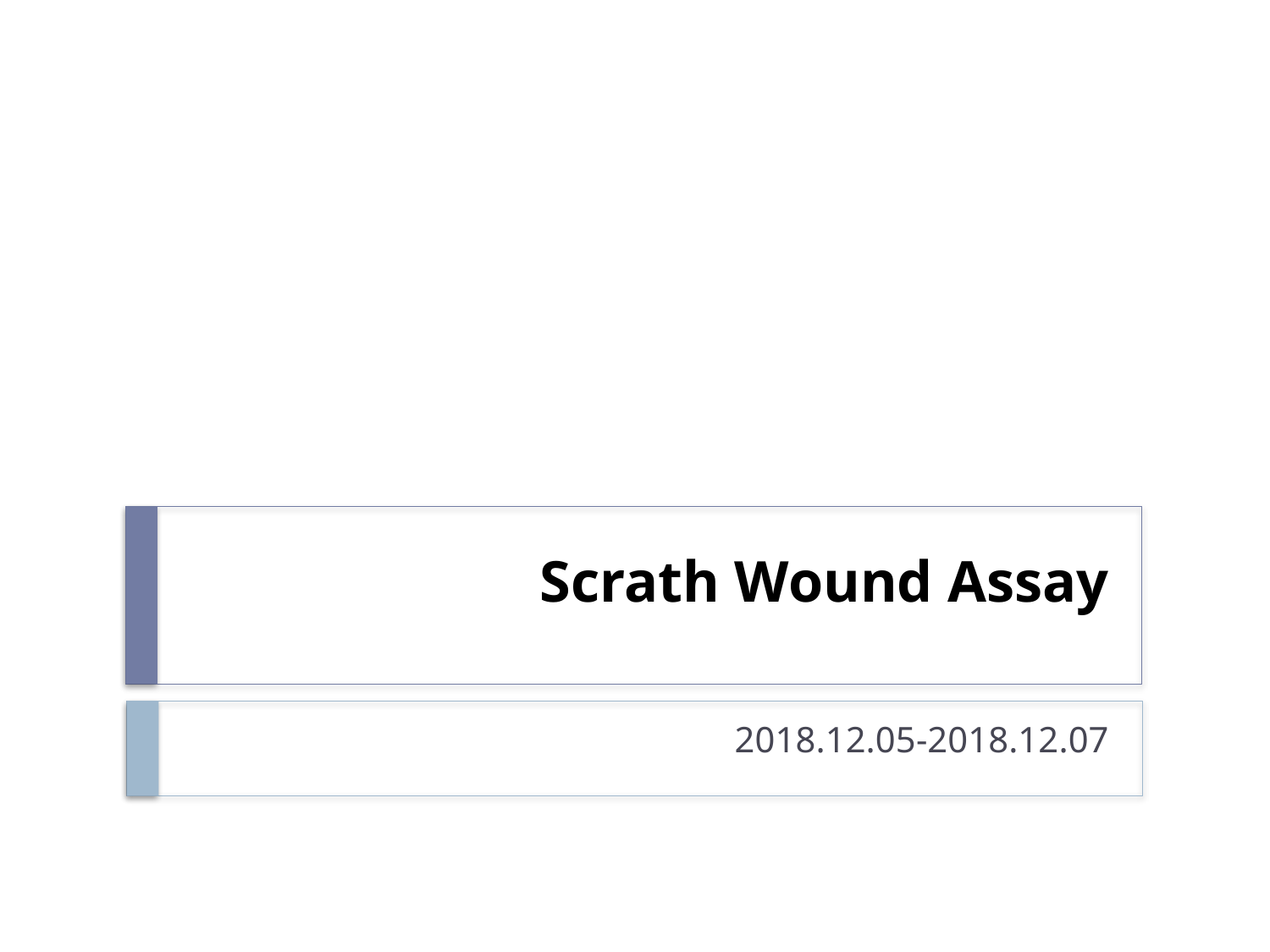

# Scrath Wound Assay
2018.12.05-2018.12.07

## Slide 2
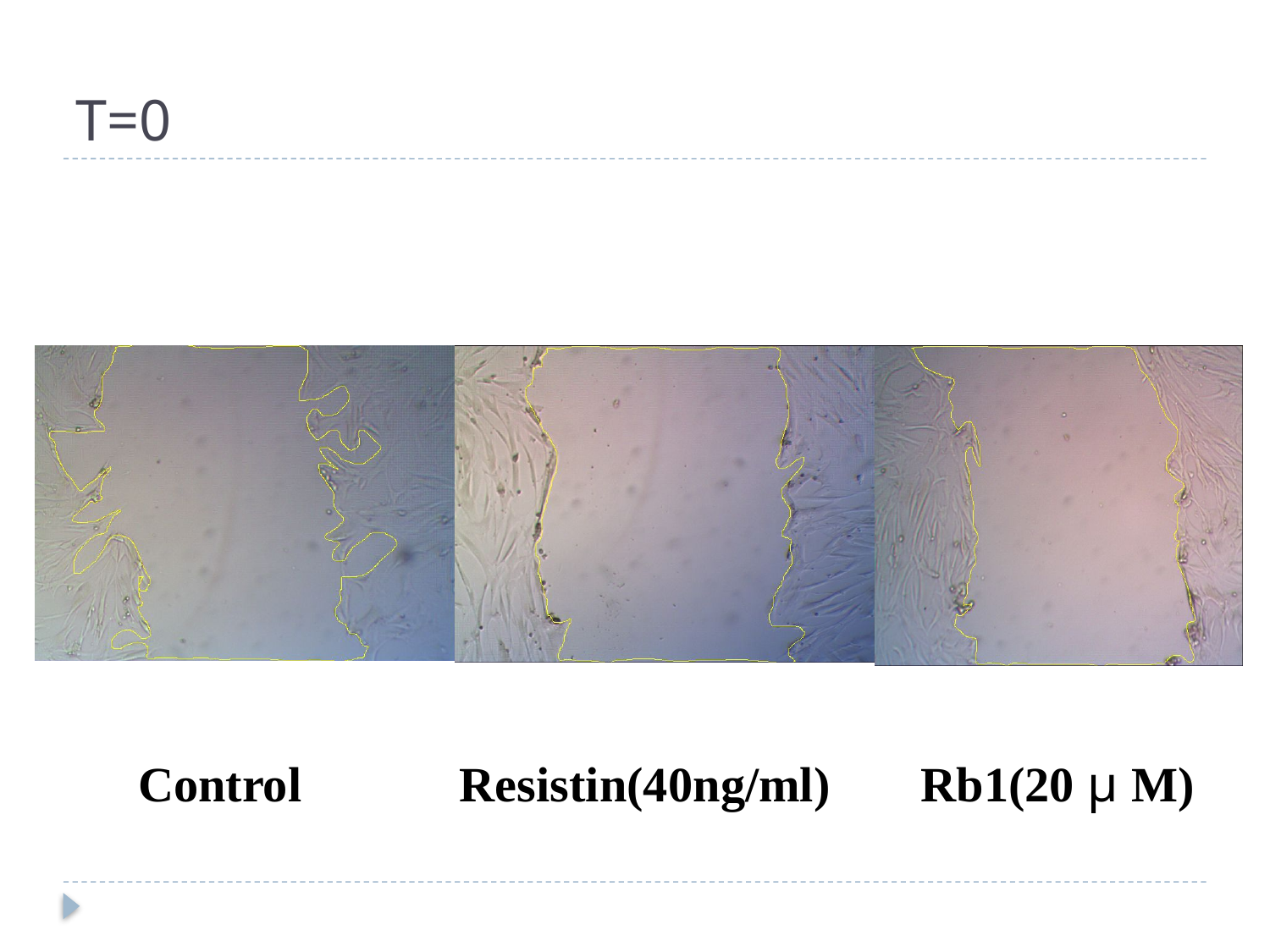

# T=0
Control
Resistin(40ng/ml)
Rb1(20 µ M)

## Slide 3
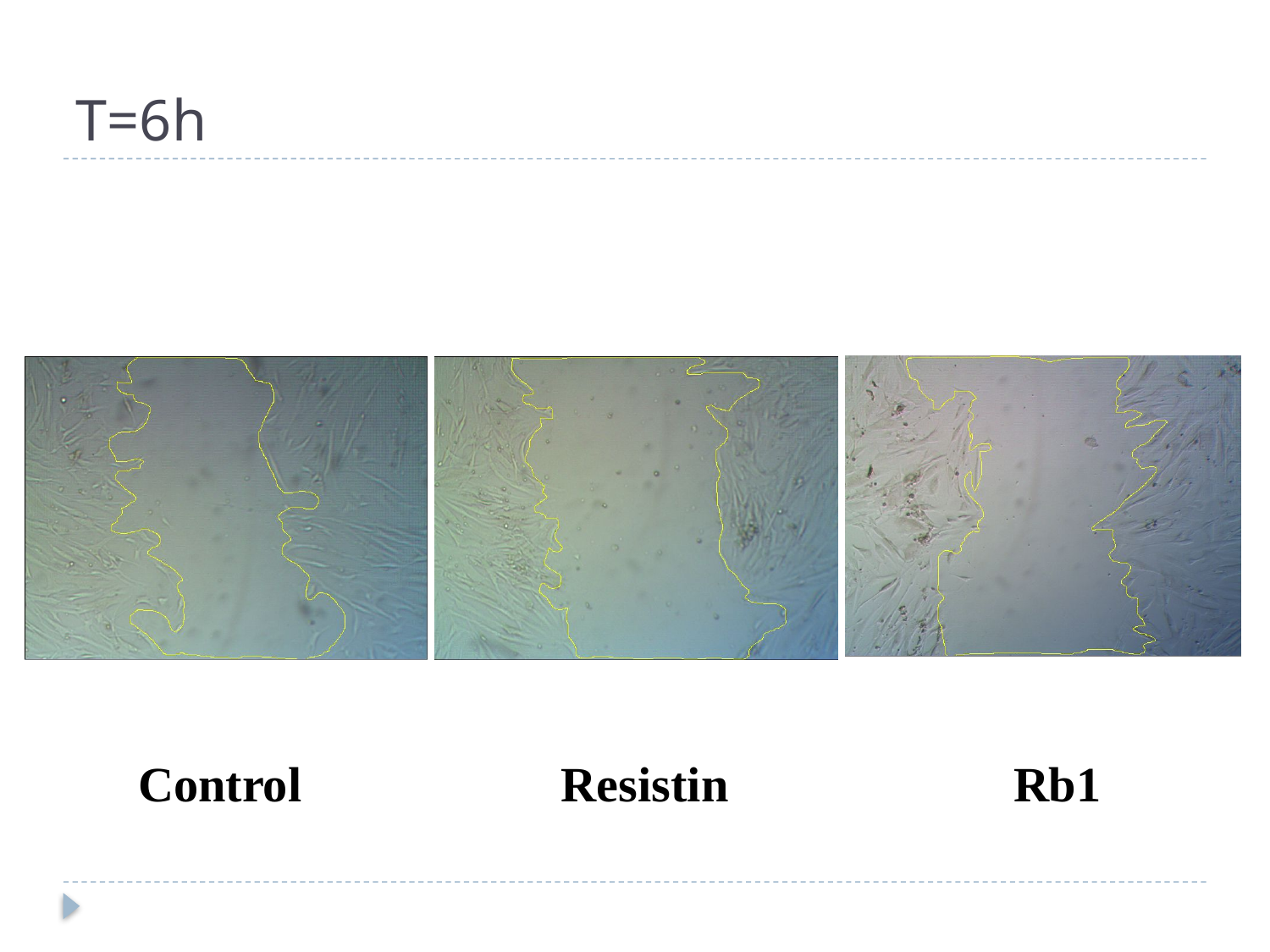

# T=6h
Control
Resistin
Rb1

## Slide 4
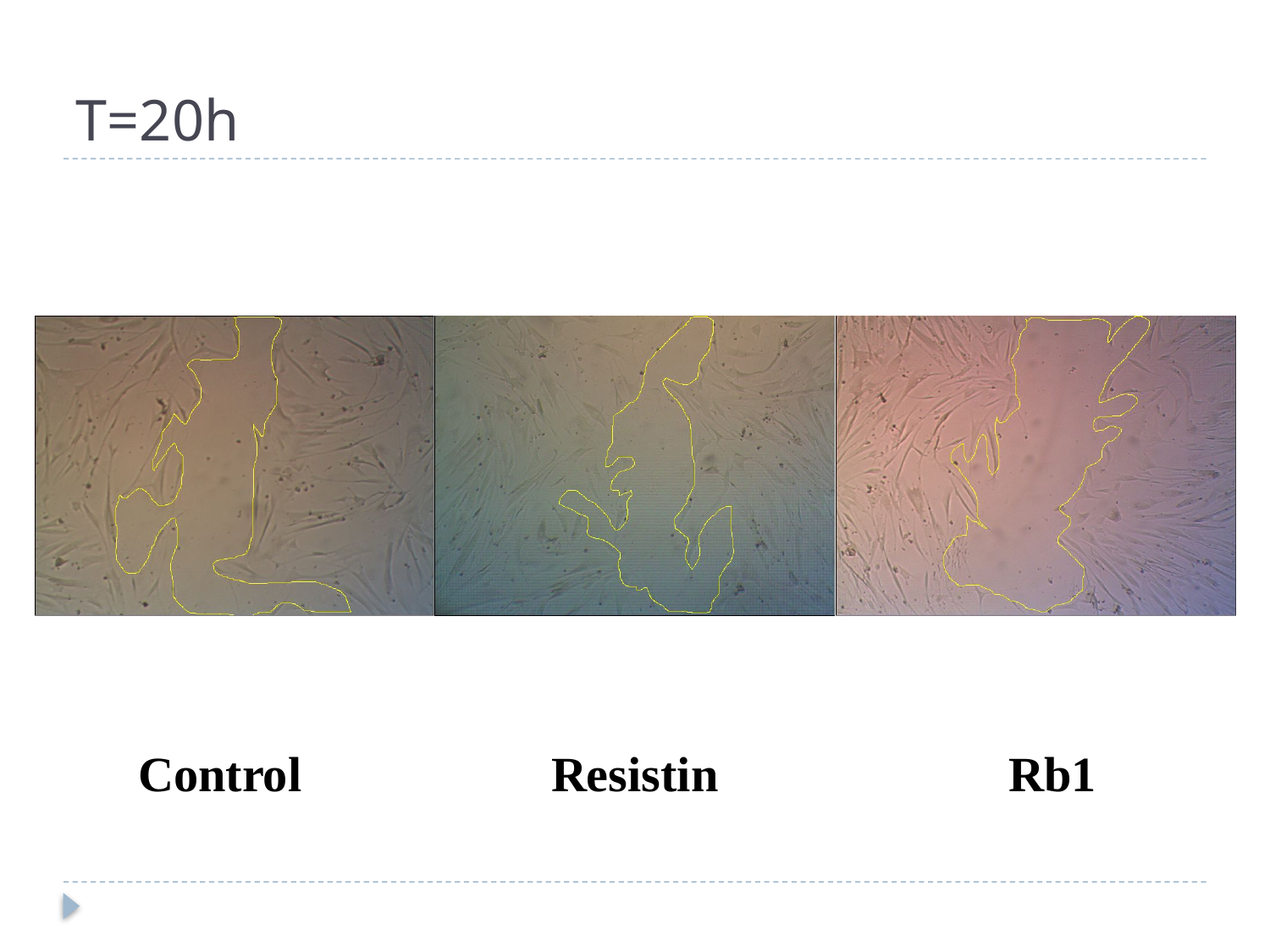

# T=20h
Control
Resistin
Rb1

## Slide 5
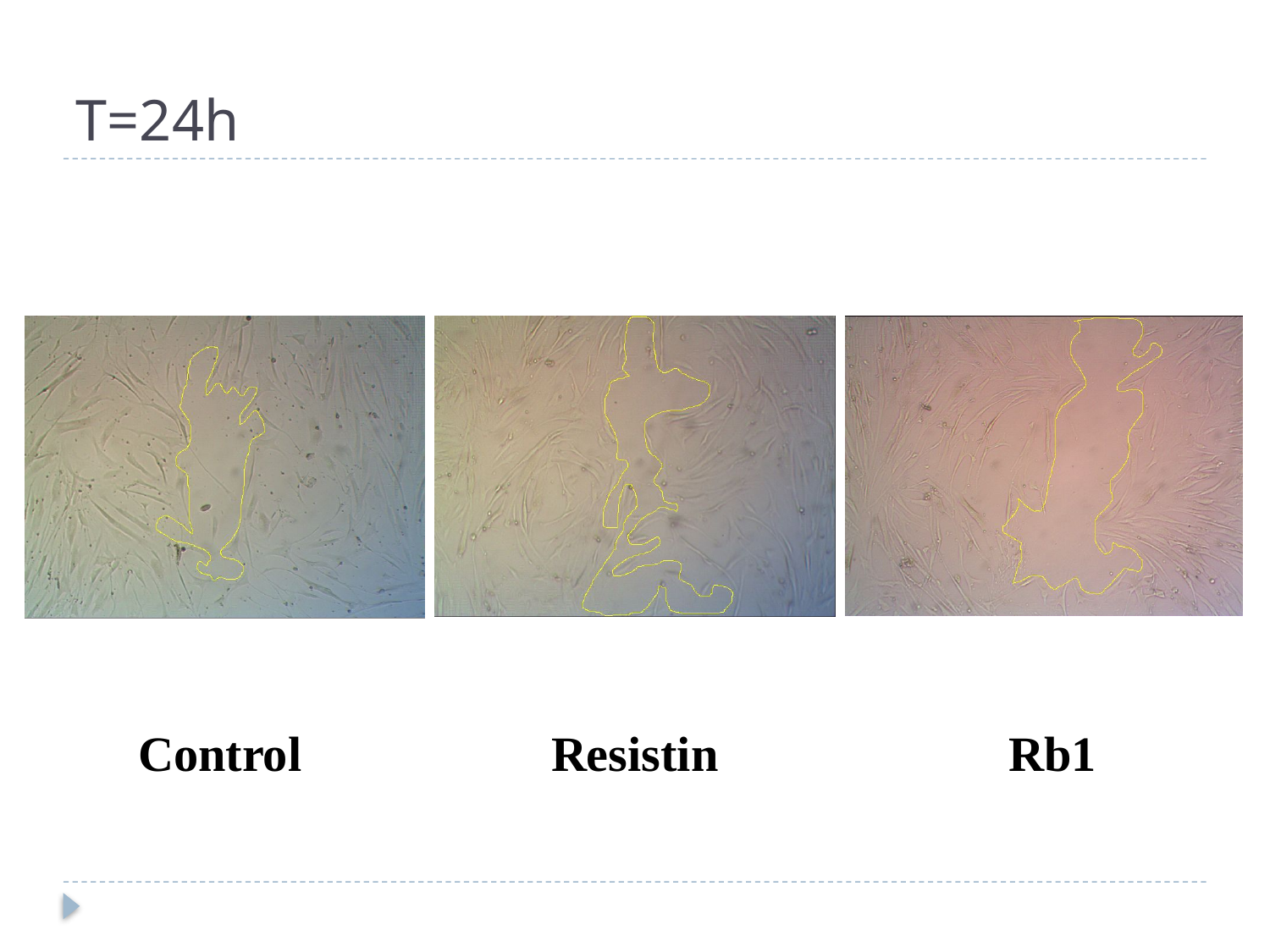

# T=24h
Control
Resistin
Rb1

## Slide 6
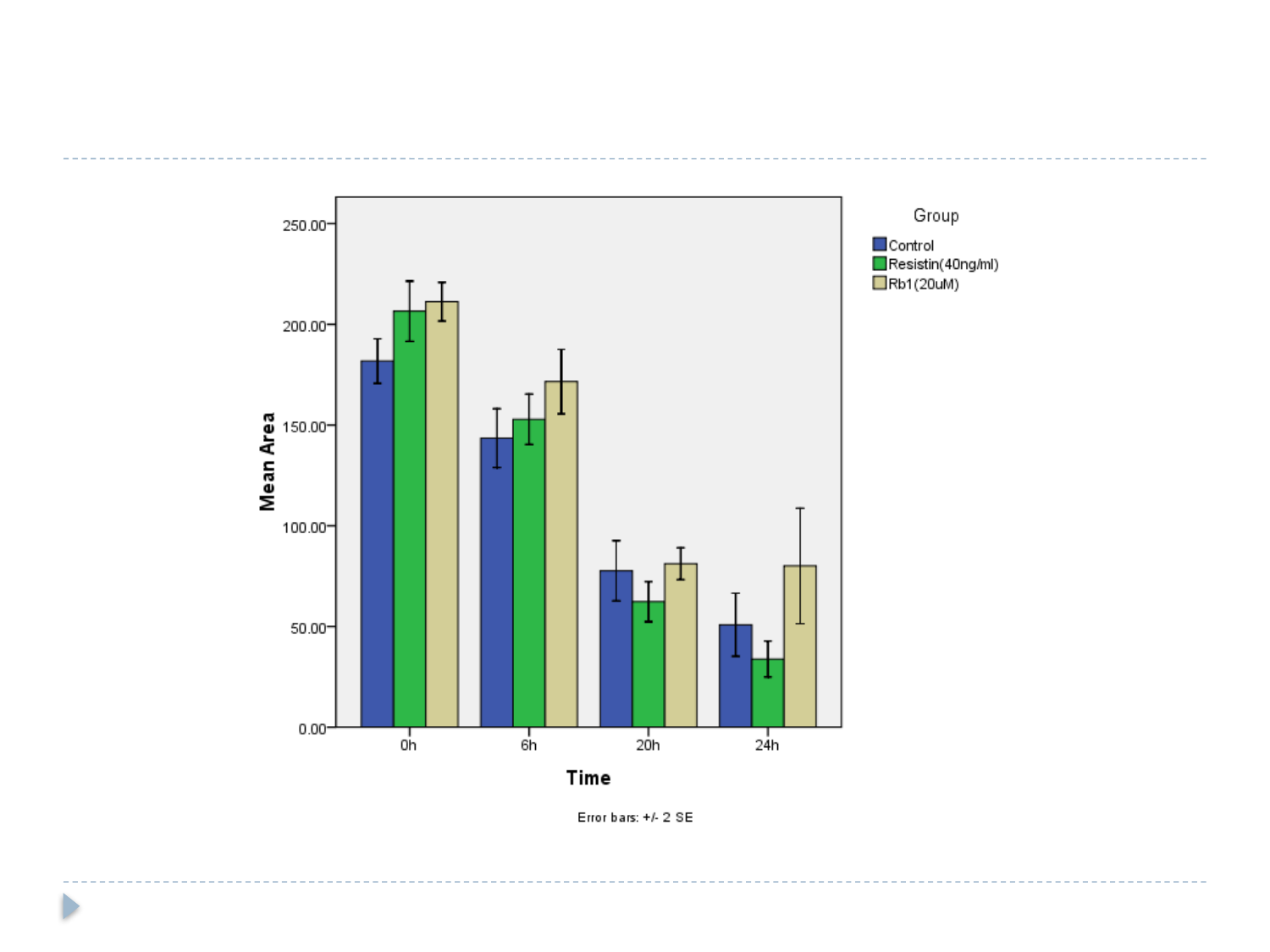

#

## Slide 7
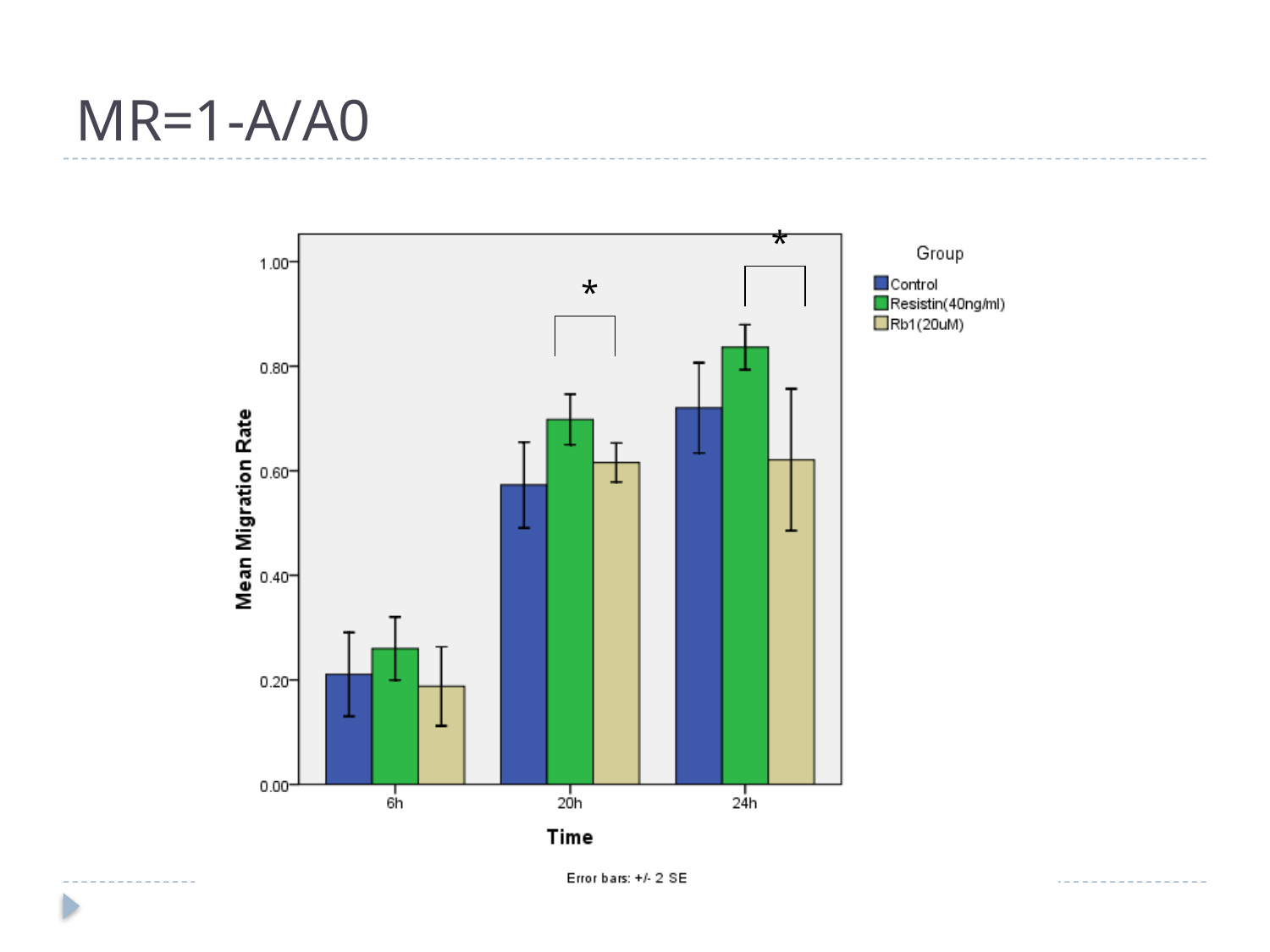

# MR=1-A/A0
*
*
