# Supplementary material for: Ginsenoside Rb1 protects human vascular smooth muscle cells against resistin-induced oxidative stress and dysfunction
Source: Front Cardiovasc Med. 2023 May 25;10:1164547. doi: 10.3389/fcvm.2023.1164547 (PMC10248054; doi:10.3389/fcvm.2023.1164547)
Supplement: Supplementary file 1 [file Datasheet1.zip › Raw data/Fig 5 SOD/SOD ACTIVITY-2.pptx]

## Slide 1
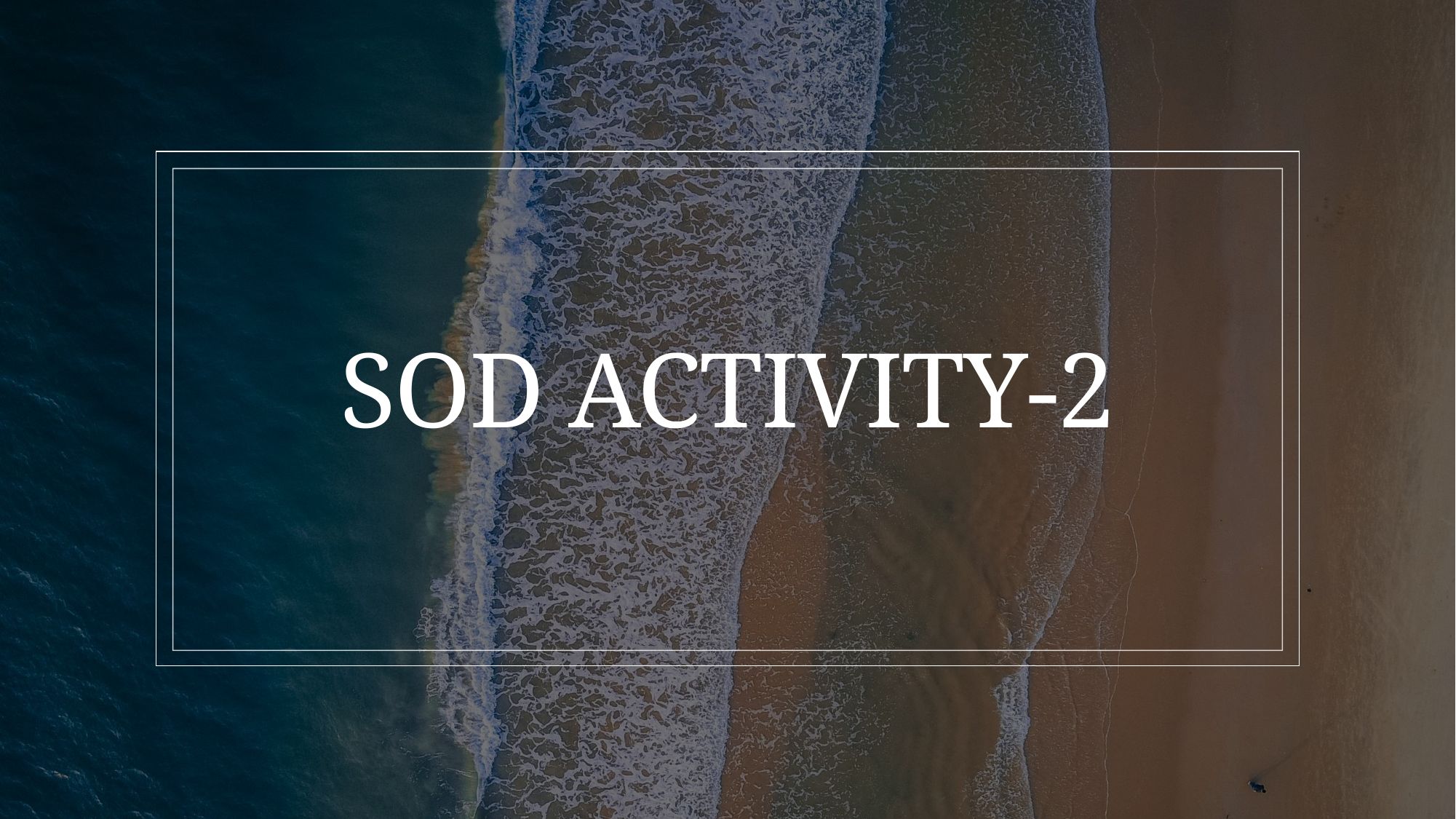

# SOD ACTIVITY-2

## Slide 2
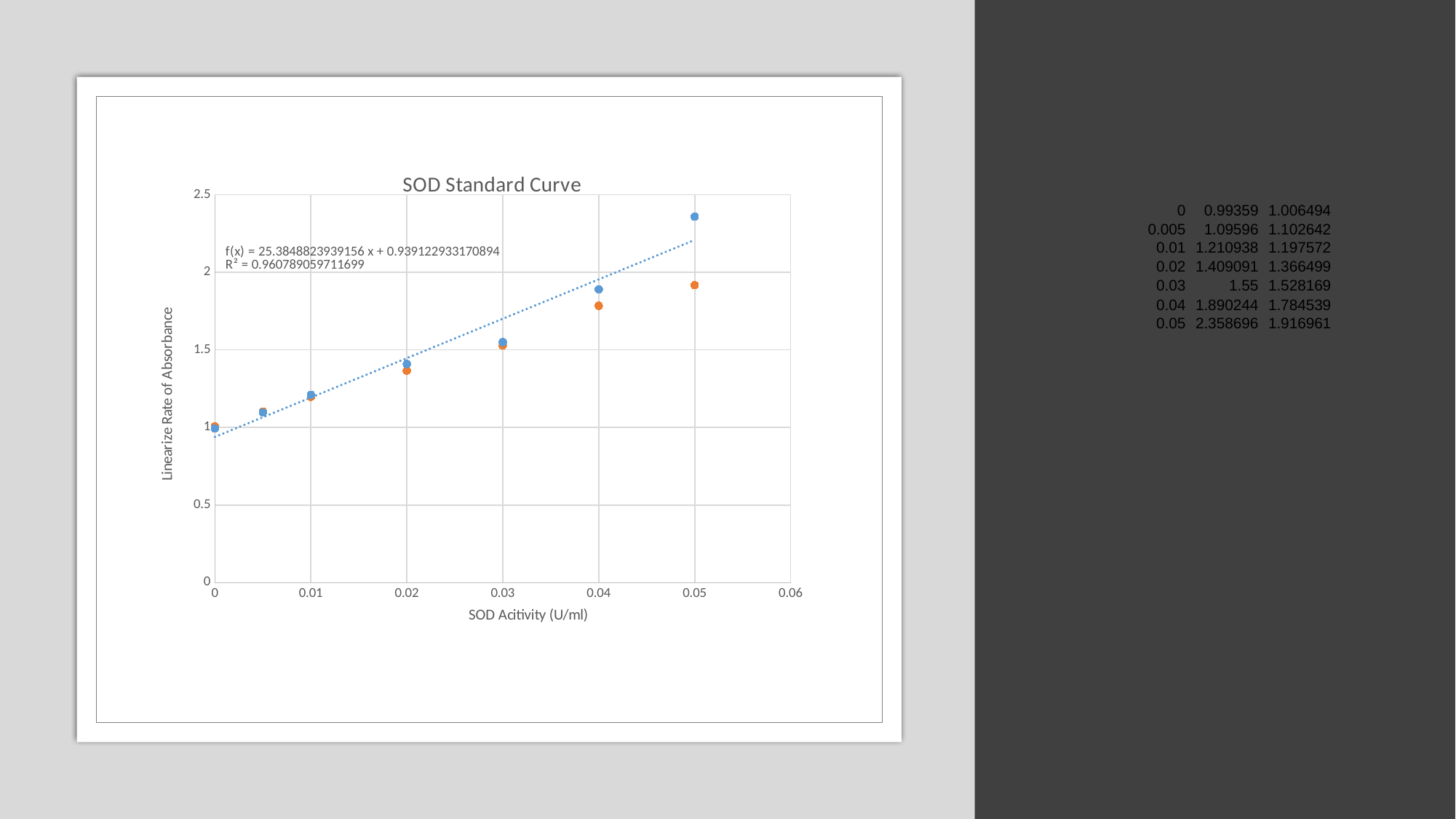

#
### Chart: SOD Standard Curve
| Category | | |
|---|---|---|| 0 | 0.99359 | 1.006494 |
| --- | --- | --- |
| 0.005 | 1.09596 | 1.102642 |
| 0.01 | 1.210938 | 1.197572 |
| 0.02 | 1.409091 | 1.366499 |
| 0.03 | 1.55 | 1.528169 |
| 0.04 | 1.890244 | 1.784539 |
| 0.05 | 2.358696 | 1.916961 |

## Slide 3
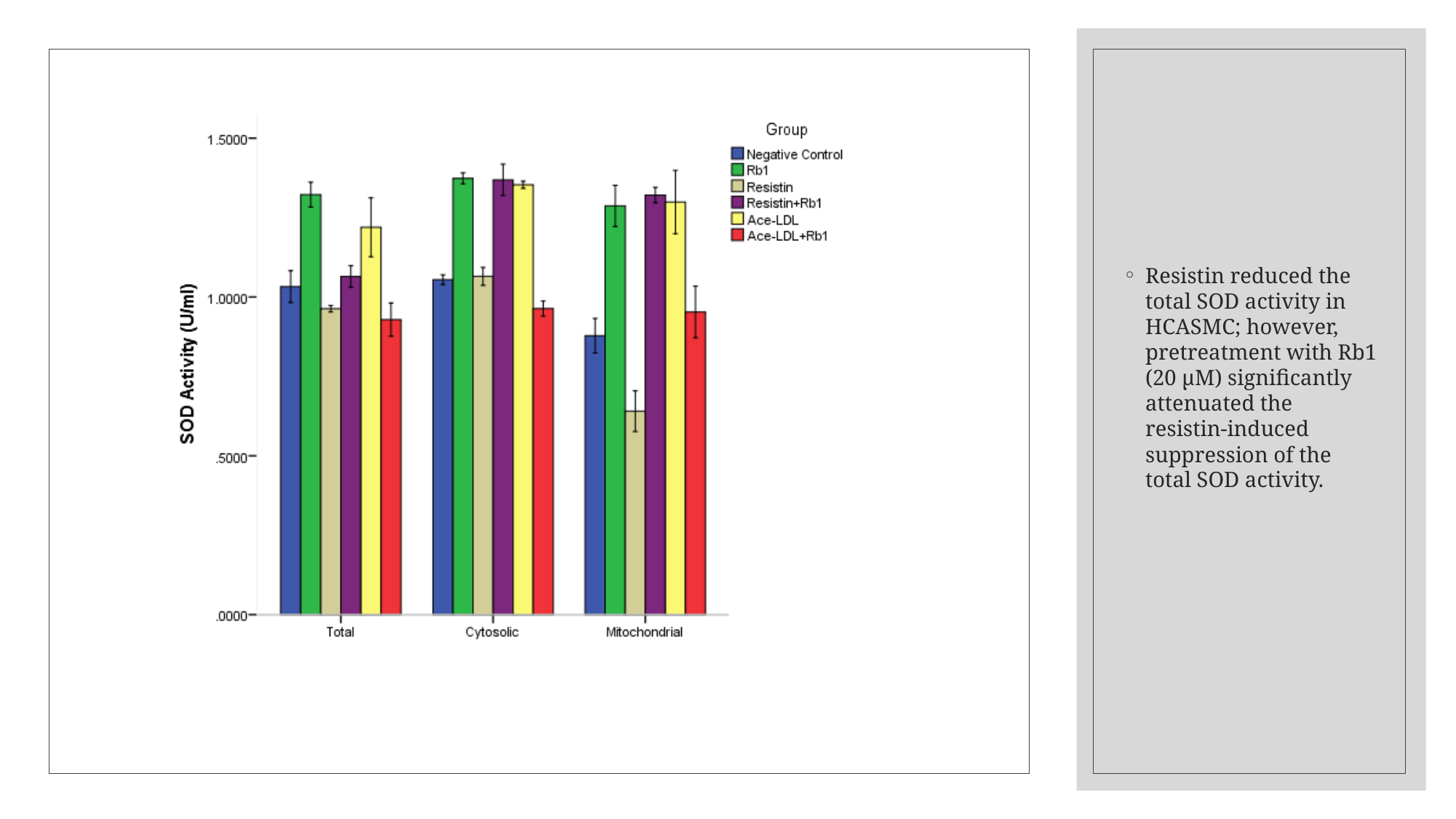

#
Resistin reduced the total SOD activity in HCASMC; however, pretreatment with Rb1 (20 µM) significantly attenuated the resistin-induced suppression of the total SOD activity.

## Slide 4
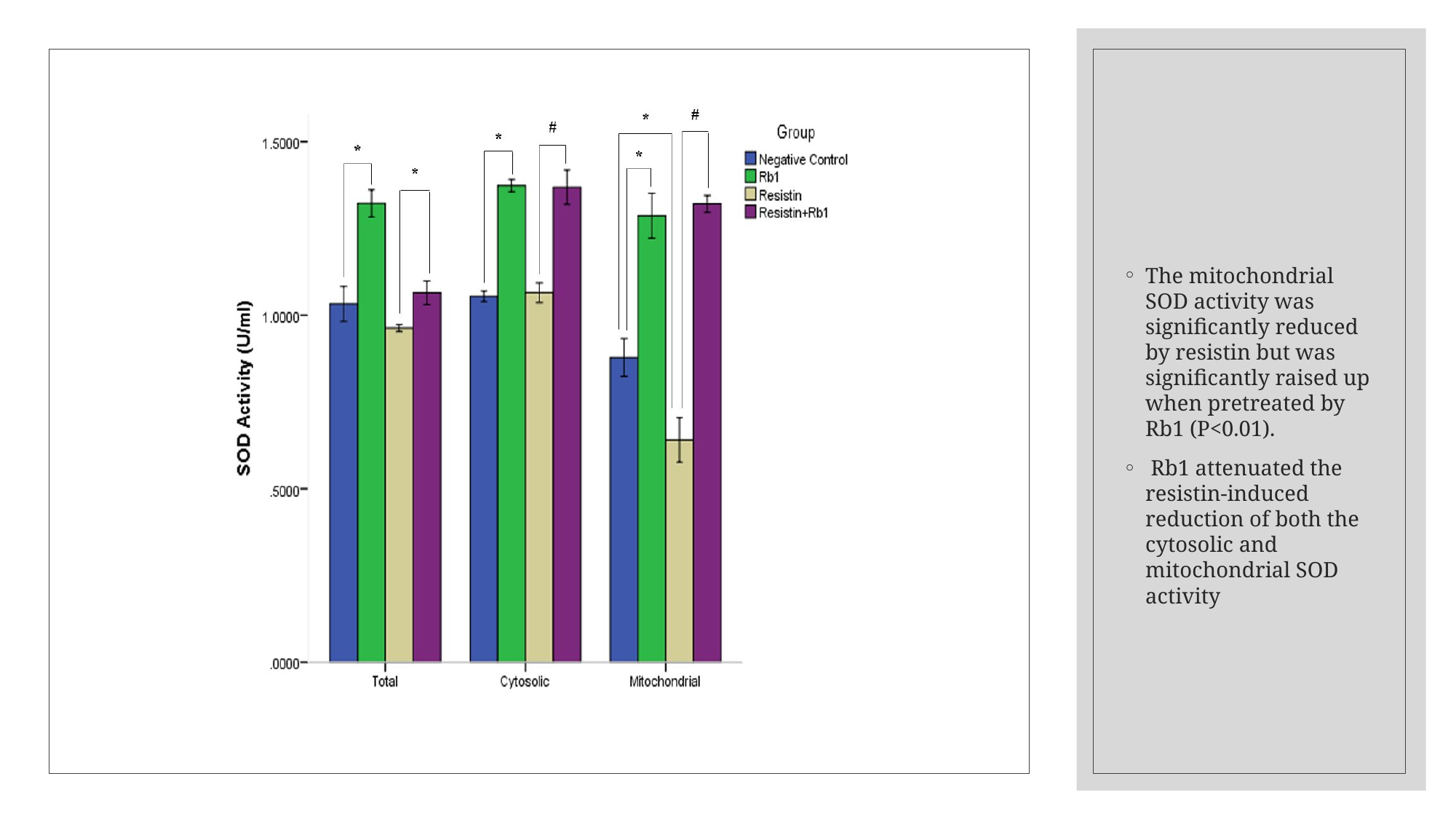

#
The mitochondrial SOD activity was significantly reduced by resistin but was significantly raised up when pretreated by Rb1 (P<0.01).
 Rb1 attenuated the resistin-induced reduction of both the cytosolic and mitochondrial SOD activity
