# Supplementary figures and images for: Ginsenoside Rb1 protects human vascular smooth muscle cells against resistin-induced oxidative stress and dysfunction
Source: Front Cardiovasc Med. 2023 May 25;10:1164547. doi: 10.3389/fcvm.2023.1164547 (PMC10248054; doi:10.3389/fcvm.2023.1164547)

## Slide 1
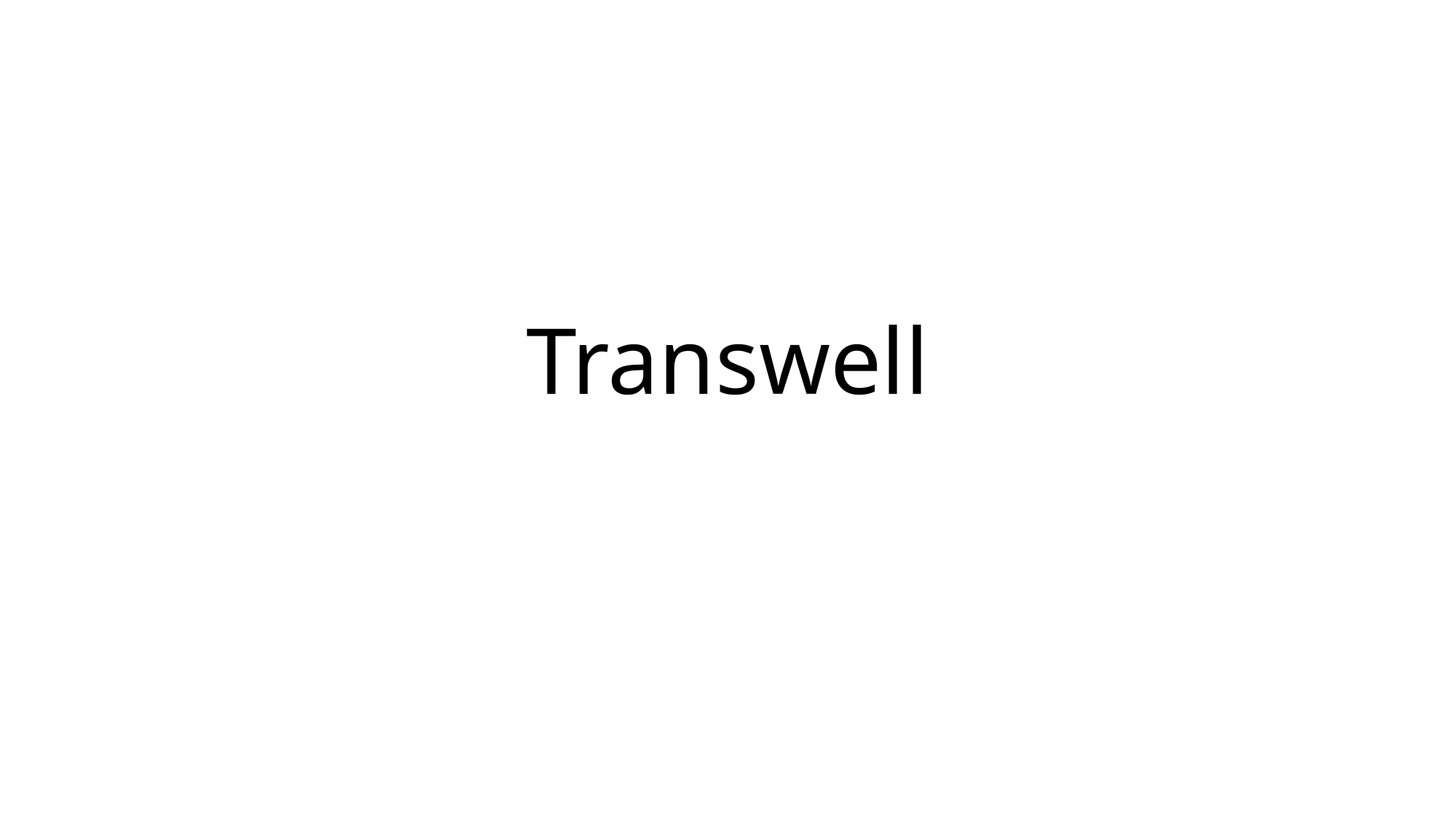

# Transwell

## Slide 2
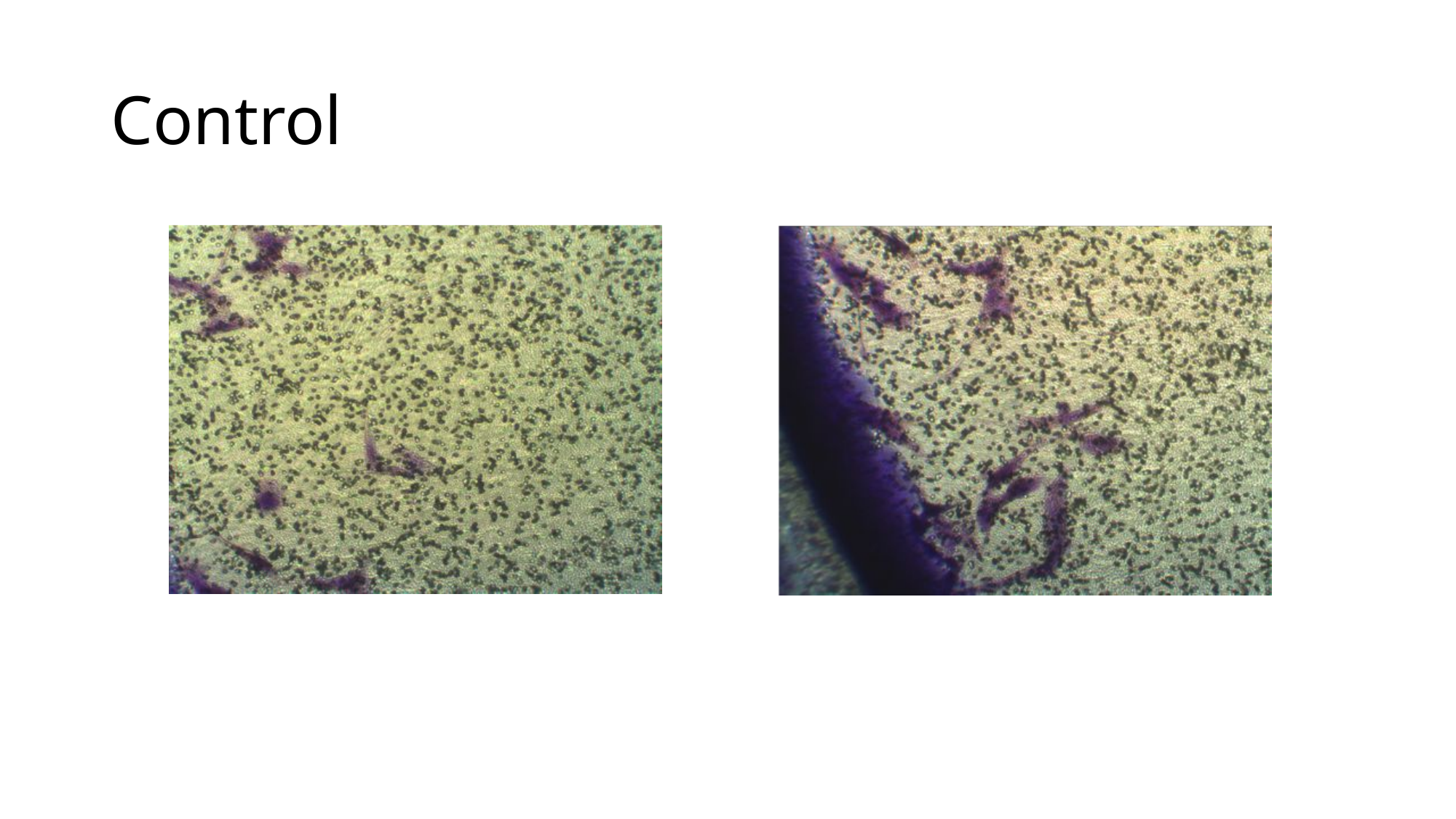

# Control

## Slide 3
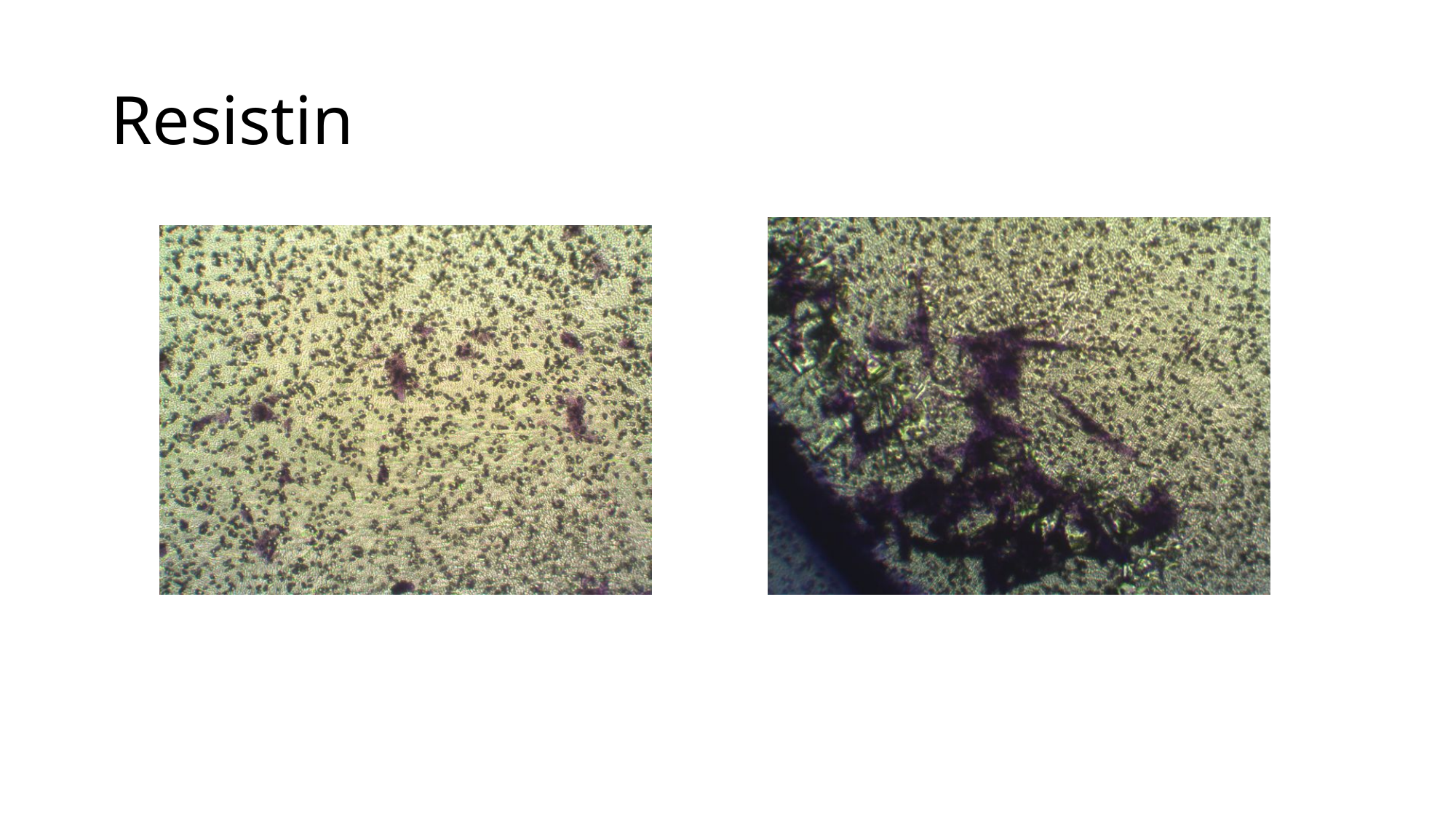

# Resistin

## Slide 4
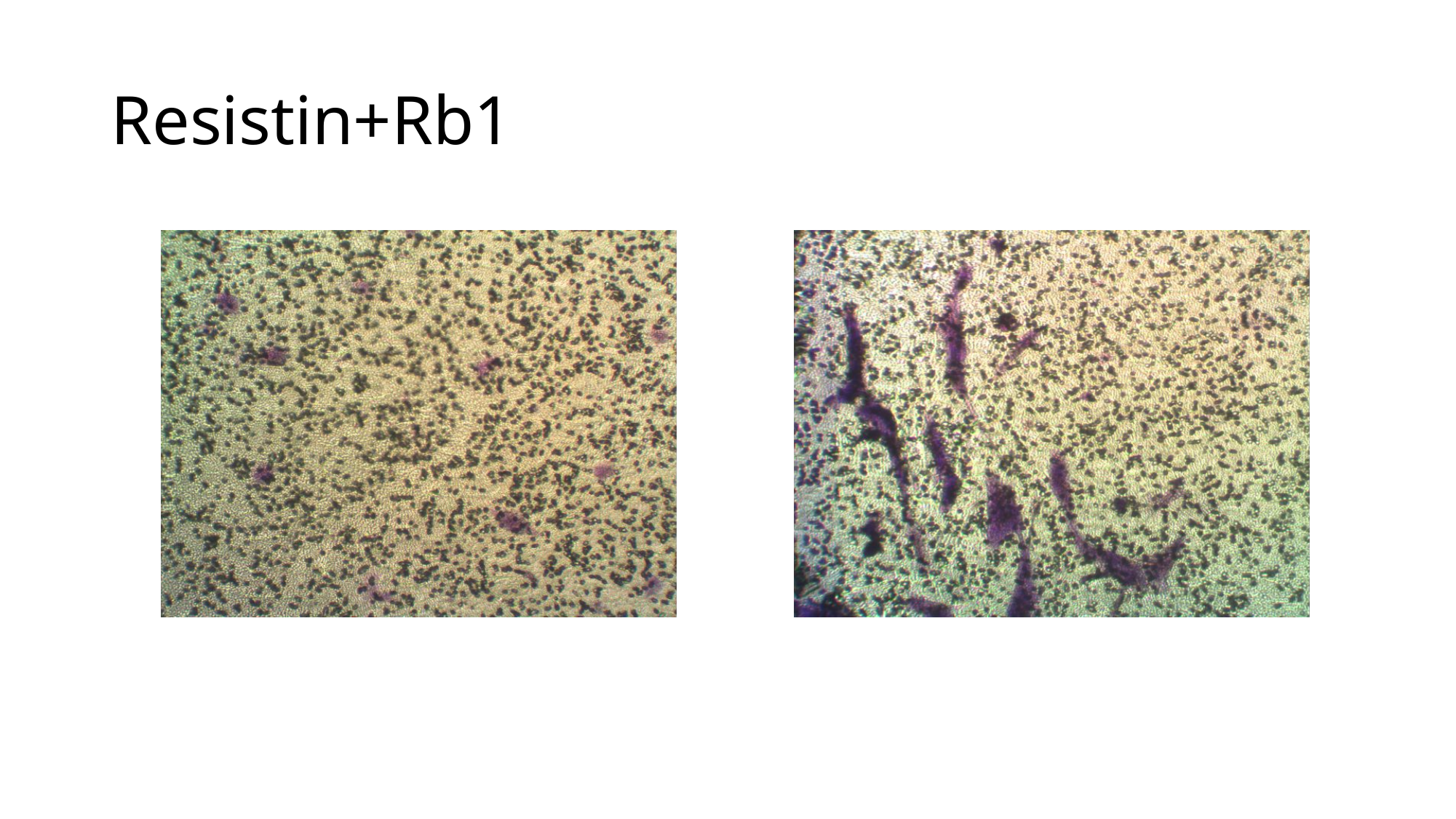

# Resistin+Rb1

## Slide 5
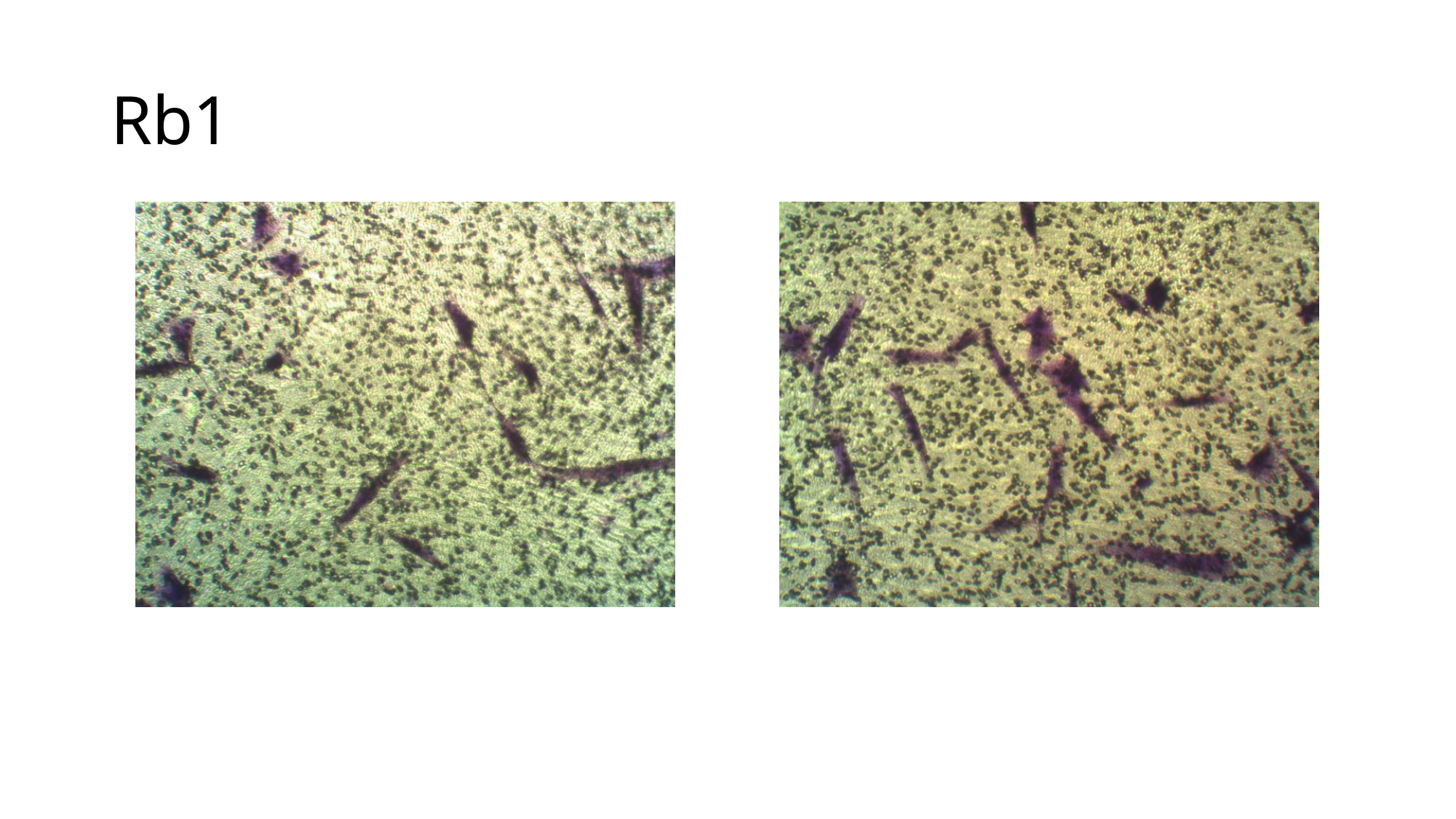

# Rb1

Supplement: Supplementary file 1 [file Datasheet1.zip › Raw data/Fig 2 Transwell/Transwell.pptx]
