# Supplementary material for: Ginsenoside Rb1 protects human vascular smooth muscle cells against resistin-induced oxidative stress and dysfunction
Source: Front Cardiovasc Med. 2023 May 25;10:1164547. doi: 10.3389/fcvm.2023.1164547 (PMC10248054; doi:10.3389/fcvm.2023.1164547)

Intracellular ROS were evaluated using the fluorescent probe 2′,7′-dichlorodihydrofluorescein diacetate (H2DCFDA) in a 96-well plate (2.5×104/well). The fluorescence of the cell homogenate was measured on a fluorescence plate reader at Ex/Em=485/535nm at the end point. Visual confirmation of changes in ROS with different treatment group can be found in the raw data provided. They are as follows.


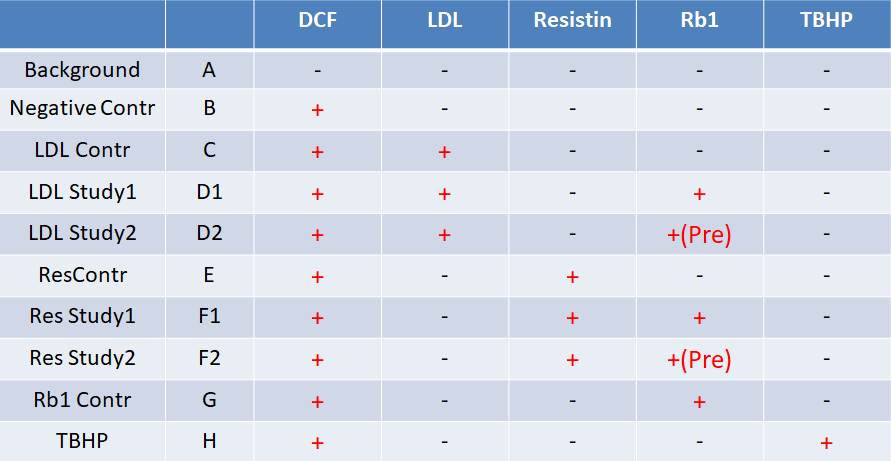


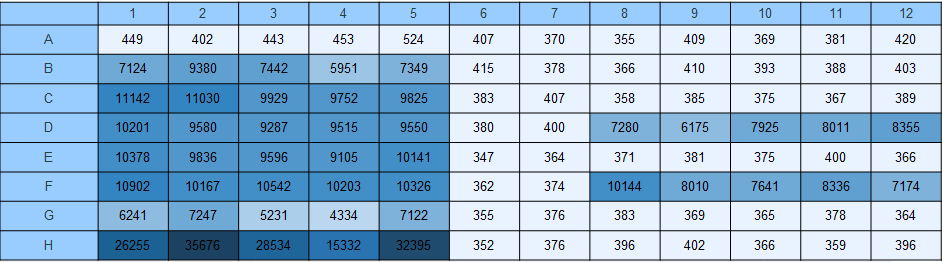


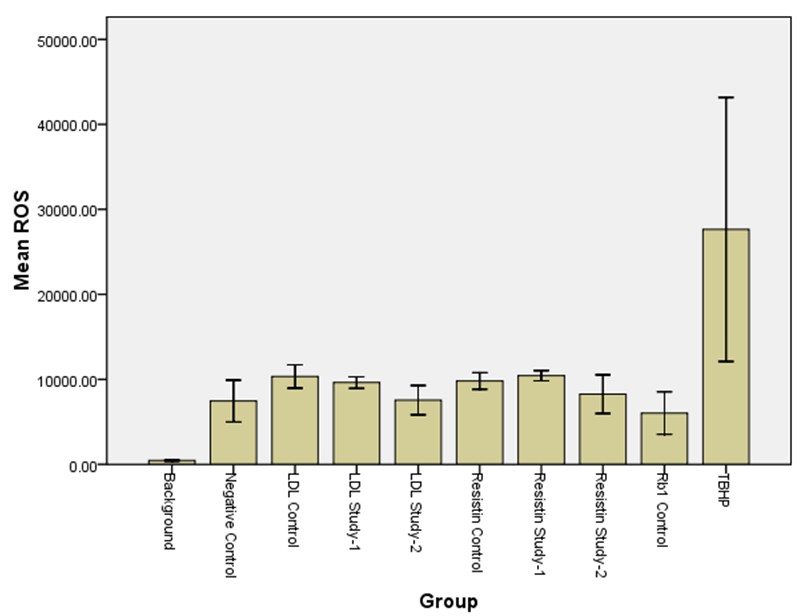

Supplement: Supplementary file 2 [file Table2.docx]
